# Recent Advances in Silk Fibroin Derived from *Bombyx mori* for Regenerative Medicine

**DOI:** 10.3390/jfb17010012

**Published:** 2025-12-24

**Authors:** Yuhao Zhang, Iman Roohani

**Affiliations:** Lab of Advanced Biomaterials and Fabrication, School of Biomedical Engineering, Faculty of Engineering and Information Technology, University of Technology Sydney, Sydney, NSW 2007, Australia; zhangyuhao5426@hotmail.com

**Keywords:** silk fibroin, tissue regeneration, biomaterials, regenerative medicine

## Abstract

*Bombyx mori* silk fibroin (BMSF) has developed from a textile fibre into a mature biomaterial with broad utility in regenerative medicine, owing to its unique hierarchical molecular structure. Its excellent biocompatibility, tuneable mechanical properties, optical property, and controllable biodegradability arise from its protein conformation, which can be precisely regulated through processing and fabrication strategies. Recent advances in bioengineering have further expanded the capabilities of BMSF, enabling the development of biomaterials with engineered architectures, tailored microtopographies, and enhanced bioactivity. These technological developments have facilitated the design of scaffolds that more effectively guide tissue regeneration and enhance functional outcomes. Such constructs have demonstrated promising outcomes in the regeneration of bone, cartilage, vascular, neural, corneal, and skin tissues. This review summarises current progress while emphasising emerging trends that couple BMSF’s unique molecular features with immune-responsive design, instructive microarchitectures that guide cell behaviour, composite scaffold design, and functionalisation with bioactive molecules. BMSF has been positioned as a structurally adaptable and biologically instructive platform whose continued progression will depend on integrating advanced fabrication, mechanistic understanding, and translational standardisation.

## 1. Introduction

Several species of silkworms produce silk, and its unique biological and mechanical properties are primarily derived from the hierarchical structure of silk proteins.

The domesticated silkworm *Bombyx mori* produces the world’s most studied and commercially significant silk. Whilst this species dominates the market, other silkworms, including *Antheraea mylitta* (Tussah), *Antheraea assamensis* (Muga), and *Philosamia ricini* (Eri), yield silks with distinct properties worth examining. All these species spin cocoons from just two proteins: fibroin, which forms the structural core, and sericin, which acts as a protective coating that binds the fibroin fibres together.

**Figure 1:**
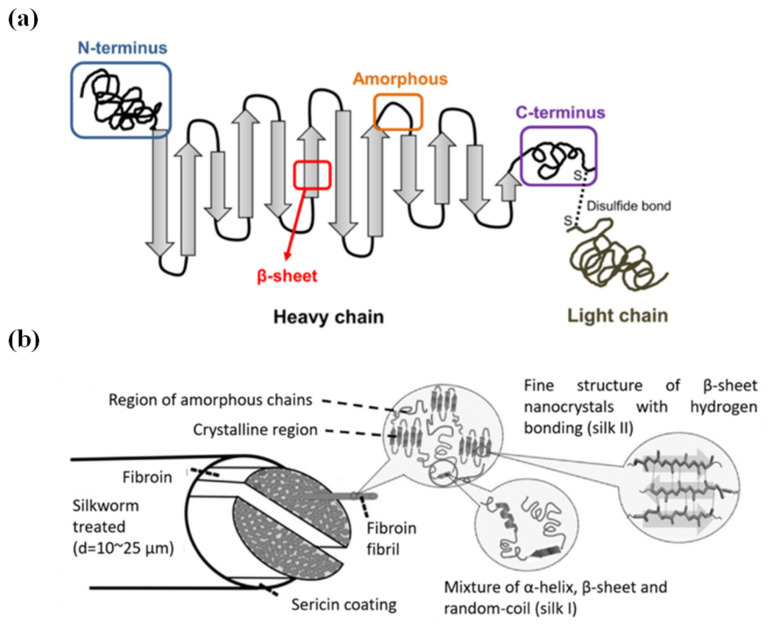
Schematics of BMSF molecular structure. (**a**) A diagram of the BMSF molecule showing the disulfide bonding between the light chain (L-chain) and heavy chain (H-chain). Reproduced from. Reprinted from Ref. [5] under the Creative Commons Attribution (CC BY 4.0) license. Copyright 2018. (**b**) A diagram showing the composition of the BMSF fibril. Reprinted with permission from Ref. [4]. Copyright 2015, John Wiley and Sons.

*Bombyx mori* silk fibroin (BMSF) also dominates biomedical applications among other silk species. It comprises three distinct components: a 350 kDa heavy chain (H-chain), a 26 kDa light chain (L-chain) linked via a disulfide bond, and a 30 kDa glycoprotein called P25, which connects to the heavy chain via a hydrogen bond [1,2,3,4,5] (Figure 1a).

The H-chain architecture reveals a striking pattern consisting of repetitive and linker domains that collectively form a (Gly-X)n motif dominated by glycine, alanine, serine, and tyrosine residues [6,7,8,9]. This composition results in a highly hydrophobic structure, explaining the water insolubility of BMSF [2]. In contrast, the L-chain adopts an amorphous structure rich in polar residues, providing hydrophobicity and making it central to BMSF reactivity and functionalisation [9,10]. This structural dichotomy enables selective modification: preserving hydrophilic L-chains maintains reactivity for further functionalisation, whilst retaining hydrophobic H-chains enhances mechanical strength. The P25 glycoprotein, though hydrophobic like the H-chain, plays a temporary yet vital role [11]. Within the silk gland, P25 stabilises the H-chain and ensures proper folding [1] but dissociates during fibre extrusion, acting essentially as a molecular chaperone.

**Figure 2:**
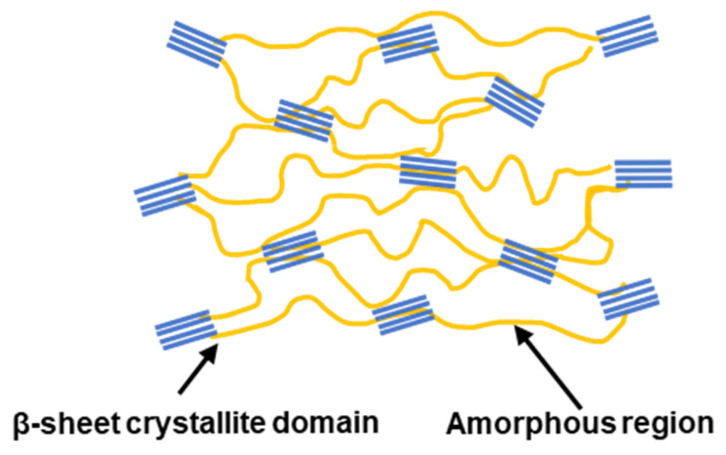
Illustration showing β-sheet crystallites (H-chain) embedded within amorphous domains (L-chain).

The H-chain’s distinctive amino acid composition drives its secondary structure formation. Glycine, lacking side chains, permits tight packing, whilst alanine’s methyl groups interact through van der Waals forces to promote β-strand formation. These β-strands align to form predominantly antiparallel β-sheets (2:1) [8,12,13]. These β-sheets stack into lamellae, with Van der Waals forces between alanine residues driving the crystallinity that defines silk’s mechanical properties [7] (Figure 1b and Figure 2). The amorphous regions contain α-helices alongside random coils. These right-handed helices form through intramolecular hydrogen bonding, where each carbonyl group bonds with the amino group four residues downstream (*n* + 4), creating a stable three-dimensional spiral [14,15].

Non-mulberry silk fibroins diverge fundamentally from BMSF’s three-protein structure, lacking the H-chain, L-chain, and glycoprotein configuration [16]. Instead, each species produces distinct, simpler structures: Tasar silk contains polymeric dimers of 197 kDa subunits, Eri silk comprises 97 kDa and 45 kDa fragments, whilst Muga silk consists of 220 kDa and 20 kDa components [17]. Despite incomplete sequence characterisation, these fibroins share BMSF’s glycine-alanine richness but contain extensive (Ala)n repeats that increase their hydrophobicity beyond that of BMSF [18].

**Figure 3:**
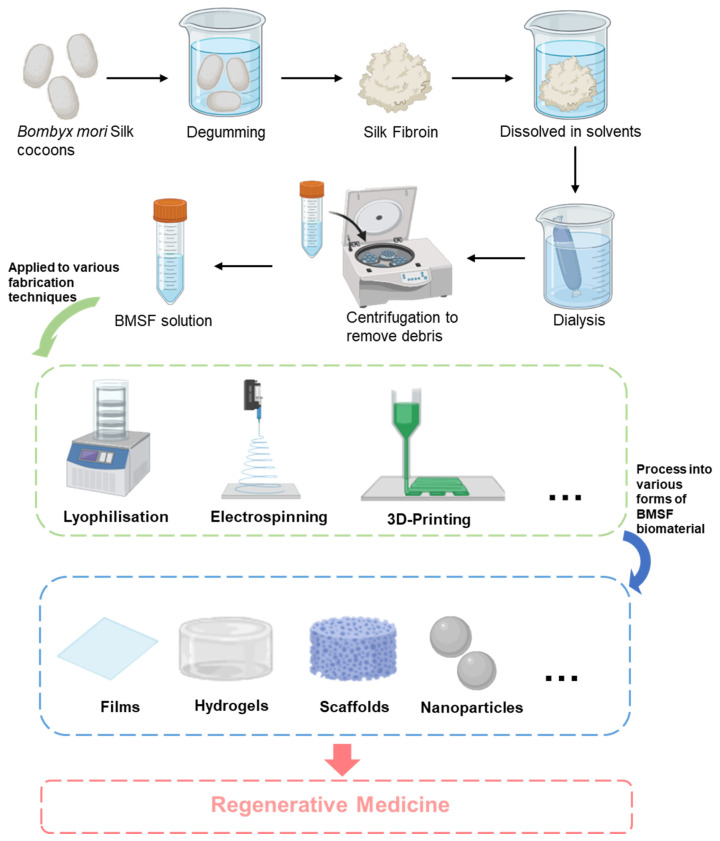
Schematic of Bombyx mori silk fibroin preparation for biomaterials. Silk biomaterials are derived from silkworm cocoons, from which sericin is removed via degumming. The fibroin is then solubilised, and toxic solvents are removed through dialysis. The resulting silk solution is further cations in regenerative medicine.

Compared to non-mulberry silk, the hierarchical structure of BMSF facilitates its processing into a versatile biomaterial suitable for tissue engineering applications. Processing begins with the extraction of BMSF, typically involving a degumming process to remove sericin. The insoluble fibroin is then dissolved in solvents such as lithium bromide, followed by dialysis and centrifugation for purification [19]. These steps yield a purified fibroin solution that can be further processed using multiple fabrication strategies, involving 3D printing, electrospinning, lyophilisation, freeze drying, and others, resulting in films, scaffolds, hydrogels, nanoparticles, and microspheres, each suited for specific regenerative medicine purposes [20] (Figure 3). Notably, recent hydrogel engineering advancements have further highlighted the emergence of smart, adaptive, and dynamically responsive BMSF systems, enhancing bioactivity and clinical translational potential of BMSF-based biomaterials [21]. BMSF can be extracted and processed into diverse biomaterial forms through standard protocols. However, the situation is more complex for non-mulberry silks due to their distinct protein architecture. Although non-mulberry silks possess RGD (Arg-Gly-Asp) sequences—integrin binding sites absent in BMSF that significantly enhance cell attachment and biocompatibility [22,23,24]—this biological advantage comes with trade-offs. Their lower molecular weight and reduced β-sheet content yield weaker but more extensible fibres than BMSF [18]. Processing poses additional challenges: the higher β-sheet content in non-mulberry sericin resists standard degumming methods, such as sodium carbonate treatment, necessitating direct gland extraction and specialised processing [17,25]. A concise comparison of the key advantages and limitations between BMSF and non-mulberry silk fibroin is summarised in Table 1.

These practical limitations explain BMSF’s dominance in tissue regeneration research. Its consistent structure, straightforward processing, and minimal batch variation make it the preferred choice for clinical applications, which is the focus of this review.

## 2. Key Properties That Make *Bombyx mori* Silk Fibroin Promising for Regenerative Medicine

### 2.1. Mechanical Properties

The mechanical strength of BMSF, including stiffness and tensile strength, is closely associated with its hierarchical molecular structure, β-sheet crystallinity, and amorphous regions, and it is dominated by the size, arrangement, and orientation of β-sheets [7,31]. Typical BMSF fibres exhibit a Young’s modulus of 10–17 GPa and an ultimate tensile strength of 300–740 MPa [7,32,33]. Compared to other natural protein-based fibres, BMSF derived from *Bombyx mori* silkworms demonstrates outstanding mechanical performance (Table 2).

The stiffness and tensile strength of BMSF are predominantly governed by β-sheet crystalline domains formed by the H-chain protein. These β-sheets are stabilised through a dense network of intermolecular hydrogen bonds and van der Waals forces, which synergistically contribute to BMSF’s remarkable mechanical properties. Studies on crosslinked BMSF hydrogels demonstrate that the transition from random coils to β-sheets significantly increases compressive stiffness [34].

The arrangement of β-sheets critically influences hydrogen bond strength. Antiparallel β-strands form shorter, linear hydrogen bonds, yielding stronger interactions, whereas parallel β-sheets exhibit longer, less stable hydrogen bonds [35]. Molecular dynamics simulations confirm this difference: antiparallel BMSF β-sheets show higher stiffness (86.8 ± 2.5 GPa) and greater rupture force required to pull central β-strands from the crystalline unit (3628 pN) compared to parallel BMSF β-sheets (27.3 ± 1.8 GPa and 2435 pN, respectively) [36]. These results indicate that antiparallel β-sheet structures are mechanically superior to parallel configurations [36].

Additionally, crystallite size is inversely proportional to fibre stiffness and tensile strength. This relationship stems from distinct deformation mechanisms: larger crystallites undergo less efficient bending deformation under stress, while smaller crystallites primarily experience shear deformation, maximising hydrogen bond efficiency [37]. BMSF’s mechanical properties arise from its complex layered structure and molecular composition, where β-sheet crystallites function as natural crosslinks that efficiently distribute and transfer mechanical stress throughout the material. Preservation of this hierarchical organisation is essential for maintaining BMSF’s exceptional mechanical performance. Since BMSF’s mechanical characteristics are principally determined by β-sheet interactions, fabrication variables can be employed to precisely tune its mechanical properties for specific applications.

**Table 2:** Comparing the mechanical data of silk fibroin derived from *Bombyx mori* to other silk species and fibrous materials.

Materials	Young’s Modulus (GPa)	Tensile Strength (MPa)	Ref.
BMSF	10–17	300–740	[7]
Tasar silk fibroin	3.7–4.1	360–400	[18]
Muga silk fibroin	4.2–4.8	470–510	[18]
Eri silk fibroin	6–6.6	540–580	[18]
Collagen	0.1–0.3	85	[38,39]
Tropoelastin	0.000003	-	[40]
Spider silk (*Néphila Clavipes*)	10.9	875	[41,42]
Chitosan	4.8	12–100	[43,44]
Elastin	0.00081	-	[45]

### 2.2. Biodegradation

BMSF biodegradation proceeds primarily through hydrolysis catalysed by proteolytic enzyme pathways, which are highly dependent on the β-sheet content, molecular weight, and the morphological format of BMSF biomaterials [46]. In vitro studies have demonstrated that various enzymes, including matrix metalloproteinases, α-chymotrypsin, protease XIV, and collagenase IA, can degrade BMSF [47]. The efficiency of enzymic biodegradation is also heavily determined by the number and location of the hydrolysis sites in the peptide chain in which protease XIV is the most aggressive enzyme reported, capable of cleaving both the amorphous and crystalline regions [48]. In contrast, other enzymes like α-chymotrypsin preferentially digest amorphous domains.

Proteolytic hydrolysis starts with the most disordered amorphous regions, comprising random coils and glycine-rich sequences, as these regions are more accessible to enzymes [49,50]. Followed by the removal of these sequences, the ordered crystalline regions become increasingly exposed. Although crystalline regions are tightly packed and confer some resistance to enzymic degradation, enzymes can slowly hydrolyse β-sheet-rich structures after prolonged exposure [49], contributing to the long term biodegradability of BMSF.

**Figure 4:**
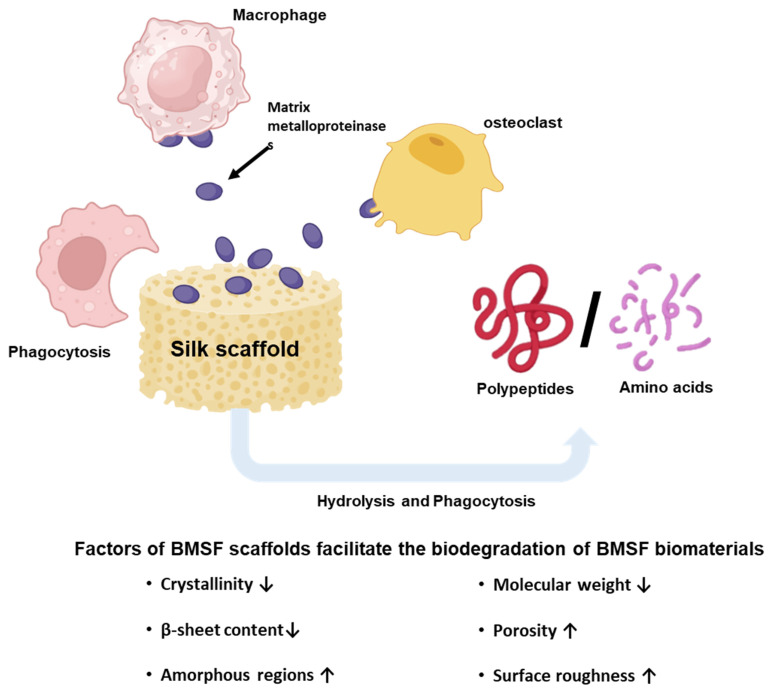
Schematic of BMSF scaffold biodegradation in vivo. Biodegradation of BMSF scaffolds is primarily mediated by macrophages and osteoclasts. These cells secrete matrix metalloproteinases to degrade silk extracellularly, while macrophages also phagocytose scaffold fragments for intracellular degradation. The mechanical and structural properties of the scaffold influence the rate of biodegradation. Arrows indicate general trends (↑ increase, ↓ decrease).

In vivo biodegradation of BMSF biomaterials is primarily driven by cellular responses, particularly an immune response [51]. Macrophages and osteoclasts play an important role in the biodegradation process. Immune biodegradation specifically involves two mechanisms: phagocytosis by macrophages and extracellular biodegradation by proteolytic enzyme secretion by cells (Figure 4) [51,52]. Matrix metalloproteinases, released by both macrophages and osteoclasts, are extensively involved in the hydrolysis of implanted BMSF [53]. Consistent with in vitro enzymatic degradation, BMSF with lower β-sheet content undergoes faster breakdown in vivo. Evidence also shows that BMSF scaffolds with greater surface exposure to immune cells, typically resulting from increased porosity, exhibited higher degradation rates than more compact architectures [48,52]. However, the implantation site can substantially alter degradation profiles, complicating the interpretation of BMSF biodegradation in vivo [54]. The biodegradation mechanisms of BMSF allow for the customisation of degradation rates through various fabrication techniques that modify scaffold properties, enabling implanted biomaterials to meet the specific clinical requirements of different tissues.

The biodegradation profile of BMSF represents a critical design parameter that can be precisely tailored to match specific end-point requirements. This tunability can be achieved through fabrication techniques that modify scaffold architecture, molecular weight, and crystallinity proportion [55,56]. The relationship between processing conditions and degradation kinetics has been well-established, with Wang et al. demonstrating that reduced molecular weight following sodium carbonate degumming accelerates degradation rates proportionally to sodium carbonate concentration [57]. This processing-dependent degradation behaviour is further exemplified by the stark contrast between natural and processed silk materials: whilst natural silk fibres exhibit minimal weight loss (5.8%), regenerated BMSF scaffolds undergo complete degradation within 14 days under enzymatic conditions [58].

Post-processing treatments provide additional control over degradation kinetics through modulation of crystalline structure. Annealing scaffolds in a vacuum with water vapour or ethanol crosslinking enhances β-sheet crystallinity, thereby retarding degradation and resorption rates [59]. Similarly, solvent selection during hydrogel preparation significantly influences longevity: Hexafluoroisopropanol (HFIP)-prepared hydrogels with reduced porosity demonstrate persistence exceeding one year in vivo, compared to 2–6 months for aqueous solvent-based counterparts [51,60]. This broad degradation window enables application-specific optimisation, where slow degradation benefits load-bearing applications such as bone defect repair requiring prolonged mechanical support, whilst rapid degradation suits transient applications including wound dressings and drug delivery systems [61]. However, achieving precise degradation kinetics that align with target tissue regeneration rates remains challenging due to complex interdependencies between graft properties and local implantation conditions.

The biocompatibility of BMSF degradation is enhanced by its glycine- and alanine-rich degradation products, which serve as biocompatible templates for protein synthesis whilst contributing to reduced immunogenicity compared to alternative biomaterials [62]. Although theoretical concerns regarding amyloid-β fibril formation and potential neurological implications have been raised, comprehensive studies confirm that BMSF degradation products remain non-cytotoxic to neural cells, reinforcing the material’s therapeutic potential across diverse regenerative medicine applications [63,64]. Understanding these degradation mechanisms is therefore critical for optimising BMSF-based tissue engineering strategies.

### 2.3. Biocompatibility

**Figure 5:**
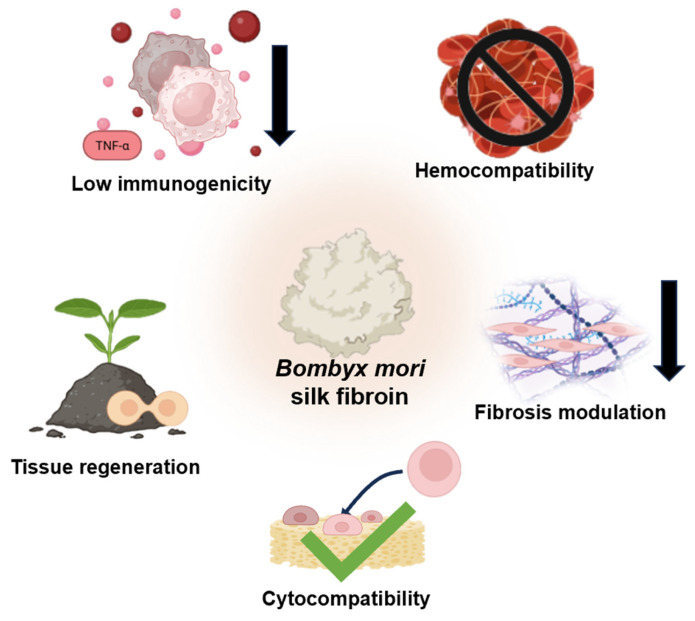
Schematic representation of the five dimensions of BMSF biocompatibility.

Biocompatibility for biomaterials can be regarded as the ability of the biomaterial to produce safe, controllable, non-toxic, and regenerative interactions with tissues and cells both in vitro and in vivo. For BMSF scaffolds, biocompatibility has the following dimensions: low immunogenicity, fibrosis modulation, favourable cytocompatibility, hemocompatibility, and capacity to support tissue regeneration (Figure 5) [65,66,67]. Immunogenicity of biomaterials plays the most vital role in determining the fate of biomaterial implants [68,69]. The foreign body response (FBR) represents an immune defence mechanism integral to wound healing, initiated upon recognition of implanted foreign materials. This complex cascade progresses from initial inflammation to late proliferative stages, mediated predominantly by innate immune cells, particularly macrophages [70]. The phenotypic polarisation of macrophages determines implantation outcomes: M1 pro-inflammatory phenotypes secrete cytokines such as tumour necrosis factor-α (TNF-α) and interleukin-1β (IL-1β), recruiting additional immune cells to form granulomas aimed at biomaterial degradation through phagocytosis and enzymatic activity [71]. Persistent foreign material presence drives the FBR transition to the proliferative phase, characterised by fibrosis and fibrous capsule formation that isolates implants from healthy tissue, ultimately compromising therapeutic efficacy. Consequently, minimising M1 macrophage polarisation represents a fundamental strategy for optimising biomaterial performance.

BMSF-based biomaterials demonstrate exceptional immunomodulatory capacity, consistently promoting M2 macrophage polarisation to establish regenerative immune environments that enhance functional outcomes [72,73,74,75]. This favourable immune profile is quantitatively supported by reduced pro-inflammatory cytokine IL-1β secretion and diminished macrophage spreading compared to synthetic polymers, including polystyrene and poly(2-hydroxyethyl methacrylate) [73]. The observed reduction in cell spreading aligns with established Toll-like receptor 4-mediated macrophage activation mechanisms involving actin rearrangement and cytokine secretion pathways [76], indicating that BMSF materials modulate fundamental immune recognition processes.

Clinical evidence across diverse tissue engineering applications substantiates BMSF’s immunomodulatory efficacy through phenotype-specific immune responses that facilitate constructive tissue remodelling. In bone regeneration applications, BMSF–hydroxyapatite composite scaffolds designed for controlled biodegradation initially induced M1 macrophage polarisation before transitioning to M2 phenotypes by day 24, supporting gradual degradation whilst promoting constructive tissue remodelling and facilitating biomaterial replacement with native tissue [72]. Similarly, electrospun BMSF nanomatrices in skin healing models demonstrated superior immunomodulatory effects compared to medical gauze, downregulating pro-inflammatory cytokines whilst upregulating regenerative transforming growth factor β (TGF-β), thereby facilitating M2 polarisation and minimising excessive inflammation to reduce cutaneous scar formation risk [74]. In vascular applications, BMSF-modified expanded polytetrafluoroethylene (ePTFE) grafts achieved superior 3-month patency rates compared to bare ePTFE [75,77], suggesting reduced neointimal hyperplasia—a primary cause of small-diameter graft chronic failure driven by excessive M1 macrophage activity [77,78].

Beyond immunomodulation, BMSF also demonstrates excellent cytocompatibility and hemocompatibility profiles, supporting robust cell regeneration, proliferation, adhesion, and biomaterial integration at implantation sites [79]. Comparative studies reveal superior pre-osteoblast (MC3T3-E1) proliferation on BMSF–hydroxyapatite composite scaffolds versus chitosan-hydroxyapatite alternatives after 3 and 5 days, indicating enhanced pre-osteoblast viability for bone regeneration applications [80]. BMSF-modified vascular grafts exhibited superior endothelial cell coverage compared to bare ePTFE grafts, essential for preventing vascular graft failure through minimised neointimal hyperplasia, thrombosis, and regulated immune responses [77]. In addition to its advantage in endothelialisation for preventing thrombosis, BMSF has also demonstrated superior hemocompatibility compared to other synthetic polymers in a full blood coagulation assay, as represented by a significantly smaller amount of blood clots on BMSF scaffolds [81]. In neural applications, BMSF scaffolds restored sciatic nerve conductivity after 6 months, achieving enhanced myelination through strong Schwann cell affinity and innervational recovery comparable to autografts [82]. These diverse examples demonstrate BMSF’s versatility as a biocompatible biomaterial capable of restoring normal tissue function by minimising FBRs whilst promoting cell regeneration across multiple organ systems.

Nevertheless, it should be noted that microarchitectural parameters, including porosity, surface roughness, and stiffness, significantly influence the immune response to BMSF. Subcutaneous implantation studies comparing electrospun BMSF scaffolds with high- and low-porosity architectures revealed that low-porosity configurations induced significantly thinner fibrotic capsules [83]. Since fibrosis is mediated by inflammation, these findings suggest that a low-porosity architecture promotes a more favourable host response with a lower immunogenicity. Surface roughness exerts additional regulatory effects on macrophage polarisation, with high-roughness BMSF films inducing thinner fibrous capsules and reduced inflammatory cytokine expression compared to low-roughness alternatives through focal adhesion kinase-Nuclear factor-κB (NF-κB) pathway modulation [84]. Scaffold stiffness represents another critical determinant of biocompatibility through macrophage polarisation regulation, with macrophages cultured on stiffer materials expressing elevated activated NF-κB levels compared to softer matrices via the piezo1-Yes associated protein (YAP) mechanosensitive pathway [85]. However, comprehensive analysis of individual mechanical property effects remains challenging due to fabrication technique limitations, where parameters such as porosity and fibre diameter exhibit proportional relationships that simultaneously influence scaffold stiffness. Consequently, fabrication techniques controlling BMSF’s mechanical and topographical features remain pivotal for optimising host responses [71]. To provide a concise overview, Table 3 highlights the main cell types mediating biocompatibility, their functional roles, and the scaffold properties that affect their responses, offering a clear reference for understanding host–material interactions.

Despite extensive preclinical evaluation, current biocompatibility assessment approaches exhibit notable limitations that constrain clinical translation. Most preclinical studies rely upon simplified two-dimensional in vitro cultures that inadequately replicate the complex cellular interactions and microenvironmental conditions of living tissues [86]. Advanced platforms, including organ-on-chip systems, organoids, and three-dimensional co-culture models, remain underutilised in routine biocompatibility testing protocols. Furthermore, predominant reliance on rodent models introduces significant limitations given substantial differences in immune responses between rodents and humans [87]. Whilst large animal models provide superior clinical relevance, cost-effectiveness constraints limit their routine implementation. Additionally, most studies focus primarily on local tissue responses whilst neglecting long-term systemic effects on distant organs and adaptive immunity development, both critical factors for predicting clinical outcomes [87]. Addressing these evaluation limitations represents an essential prerequisite for advancing BMSF-based biomaterials toward successful clinical translation.

**Table 3:** Summary of key BMSF scaffold features affecting biocompatibility of different cell types.

Cell Types	Role in Biocompatibility	BMSF Biomaterial Parameter Affecting Response	Ref.
Endothelial cells	Angiogenesis, preventing thrombosis	Surface topography, stiffness, pore size	[88,89,90]
Fibroblasts	ECM deposition, capsule formation	Stiffness, fibre alignment	[91,92]
Macrophages (M1/M2)	Fibro capsule remodelling, immune response	Scaffold porosity, stiffness, surface topography	[83,84,93,94]
Mesenchymal stem cells (MSCs)	Differentiation, regeneration	Stiffness, surface topography	[95,96,97]
Neurons	Neurite outgrowth/extension	Fibre alignment, 3D geometry, softness, biomaterial conductivity	[98,99]
Corneal stromal cells	Epithelial cell layer organisation, transparency, and integration	Surface topography, fibre alignment	[100,101]
Epidermal cells	Wound healing, reepithelialisation	Surface topography, hydrophilicity	[92,102]
Osteoblasts/Chondrocytes	Hard/soft matrix formation and integration	Stiffness, pore size	[45,103]

### 2.4. Optical Properties

BMSF exhibits exceptional optical transparency that stems from its unique molecular architecture, characterised by the combination of β-sheet crystalline domains and amorphous regions. Native BMSF films demonstrate optical transparency of 90%, whilst regenerated BMSF possesses a refractive index of 1.554 [104]. This intrinsic transparency can be attributed to the absence of aromatic amino acids tryptophan and tyrosine, which contain conjugated π electrons capable of significant UV light absorption [105]. This molecular composition enables BMSF to maintain optical clarity whilst providing the structural integrity required for biomedical applications.

The optical characteristics of BMSF are intimately linked to its secondary structure, particularly β-sheet content, which can be modulated through processing conditions. Ethanol treatment serves as an effective crosslinking strategy that reduces β-sheet content, resulting in increased transparency (>90%) and decreased optical haze through the induction of β-turns that promote more organised protein structure formation [106]. This relationship establishes β-turn enhancement as a viable strategy for producing BMSF films with superior optical clarity. Conversely, increased β-sheet crystallinity, whilst enhancing mechanical strength as previously discussed, reduces transparency due to its higher crystalline domain density that promotes light scattering and elevates the material’s refractive index [46]. This structure–property relationship provides a tuneable platform for optimising optical performance according to specific application requirements.

Architectural manipulation at the nanoscale offers additional opportunities for optical property control, enabling the simultaneous achievement of high transparency and controlled light scattering. Nanopatterned BMSF films have demonstrated exceptional performance, achieving >93% transparency with >65% optical haze, thereby enabling light diffusion without compromising optical clarity [107]. This unique combination of properties offers a significant advantage for optical coatings and devices that require controlled scattered illumination, demonstrating BMSF’s versatility in advanced photonic applications.

The combination of inherent transparency and tuneable optical characteristics positions BMSF as an attractive biomaterial for diverse implantable optical devices [108]. In ophthalmological applications, BMSF-based corneal graft films offer visible light transparency, mechanical robustness, and tuneable biodegradability [109,110], representing a superior alternative to conventional collagen-based corneal hydrogels, which often exhibit inferior swelling stability and compromised long-term transparency due to poor mechanical resilience and hydration fluctuations [111]. For implantable photonic devices such as waveguides and biosensors, BMSF’s favourable combination of mechanical properties, transparency, and processability enables direct methanol crosslinking after deposition from print heads, facilitating light delivery and detection in target tissues [112,113,114]. BMSF exhibits excellent accuracy, sensitivity, linearity, and mechanical properties whilst remaining cost-effective to fabricate.

The unique optical properties of BMSF, derived from its distinctive protein conformation and enhanced by the absence of UV-absorbing aromatic amino acids, can be precisely tuned through chemical crosslinking and architectural control. This tunability, combined with inherent biocompatibility and biodegradability, positions BMSF as a versatile alternative to conventional optical materials, offering advantages in stability, long-term transparency, and cost-effective processing whilst avoiding the immune reactivity associated with other biomaterials. These characteristics establish BMSF as a promising platform for advancing implantable optical technologies across diverse biomedical applications.

## 3. Applications of *Bombyx mori* Silk Fibroin in Regenerative Medicine

### 3.1. Cartilage

The inherently limited regenerative capacity of articular cartilage presents a significant clinical challenge in orthopaedic medicine [115]. As an avascular and aneural tissue, cartilage lacks sufficient nutrient supply and waste removal mechanisms, severely constraining its self-repair capacity [116]. Consequently, even minor injuries often fail to achieve complete healing, progressing to more extensive degenerative changes. Current treatment strategies, including autologous grafting and osteochondral grafting, have limited long-term success due to donor site morbidity and fibrocartilage formation rather than native hyaline cartilage restoration [117]. These limitations of conventional treatments have driven the development of alternative tissue regeneration strategies, which can be broadly categorised into mechanical property manipulation, surface decoration with bioactive molecules, and immunomodulation. BMSF-based composite scaffolds have emerged as particularly promising solutions (see examples in Table 4), providing both mechanical support and chondrogenic potential for cartilage repair applications.

#### 3.1.1. Silk Fibroin-Based Composite Scaffolds

Recent investigations have explored diverse BMSF-based composite scaffold for optimising cartilage regeneration.

Electrospun bilayer BMSF–polycaprolactone (PCL) composite scaffolds were fabricated with upper- and lower-layer ratios of 1:3 and 1:4, respectively, and subsequently gas foamed with carbon dioxide to generate a three-dimensional structure with defined cartilage and bone regions. When implanted into rat osteochondral defects, the bilayer scaffold significantly enhanced osteochondral repair at 12 weeks post-implantation, as indicated by increased International Cartilage Repair Society (ICRS) scores compared to 2D scaffolds and untreated controls [125]. However, this study lacked detailed characterisation of scaffold degradation and long-term mechanical stability—critical parameters for cartilage repair under load-bearing conditions. Surface mechanical properties represent another crucial design parameter, as demonstrated by BMSF hydrogels with tuneable surface rigidity ranging from 15.41 to 50.67 kPa through modulation of DNA content. Hydrogels with intermediate rigidity (30.21 kPa) exhibited optimal performance in a rat knee joint defect model, achieving cartilage recovery comparable to the sham group at 12 weeks, likely via upregulation of the transforming growth factor β-signalling pathway. Hydrogels with lower or higher rigidity showed inferior repair, highlighting the importance of precisely tuning mechanical cues to guide chondrogenic differentiation [126]. Whilst this finding underscores the importance of mechanical cues in guiding stem cell fate, the detailed cellular interactions between stem cells and the designed BMSF scaffolds remain incompletely characterised.

Three-dimensional printing technologies have enabled precise architectural control of BMSF-based scaffolds, facilitating systematic investigation of structure–function relationships. Hong et al. utilised digital light processing 3D printing using UV (365 nm) with a resolution of 30 μm for the preparation of BMSF–glycidyl methacrylate scaffolds encapsulating chondrocytes. In a rabbit tracheal defect model, the BMSF composite scaffolds supported robust cartilage formation by week 6, characterised by lacunae containing mature chondrocytes and well-organised cartilage matrix deposition [127]. However, the study did not report long-term mechanical performance or degradation kinetics of the printed constructs, which are essential parameters for clinical translation in dynamic airway environments. Similarly, injectable hydrogel formulations encapsulating chondrocytes have shown comparable outcomes [128]. Building upon 3D printing capabilities, Yan et al. fabricated BMSF and hydroxypropyl cellulose methacrylate composite scaffolds with distinctive porosity profiles, demonstrating that higher porosity scaffolds (87.10 ± 1.30%) with pore sizes ranging between 50 and 100 μm exhibited optimal rheological and structural properties than scaffolds with lower porosity. In vitro, the composite scaffold increased expression of essential cartilage differentiation factors, subsequently leading to superior cartilage formation in a rabbit knee defect model at 12 weeks compared to untreated and BMSF-only controls [129]. However, these studies lack direct comparative analyses with other bioink systems or scaffold materials, limiting contextualisation of their relative efficacy.

Translation to large animal models is essential for comprehensive preclinical evaluation given species differences in immune responses that cannot be predicted from rodents. It has been achieved through BMSF–chitosan with different concentrations of decellularised cartilage matrix (DCM) hydrogels. The composite BMSF–chitosan hydrogel with a higher concentration of DCM (20 mg/mL) achieved the best MSC chondrogenic differentiation in vitro, with markedly elevated chondrogenic differentiation markers expression compared with both DCM-free and lower DCM (15 and 10 mg/mL) concentrations. When implanted into rabbit knee defects, the 20 mg/mL DCM hydrogel produced significantly higher ICRS scores between weeks 4 and 12, with uniform hyaline cartilage deposition. Importantly, these findings were reproduced in sheep femoral condyle defects at 3 months, where the same formulation yielded superior structural restoration compared to BMSF–chitosan without DCM [130]. These promising results suggest that bioactive molecules within DCM facilitate chondrogenic differentiation beyond mechanical support alone.

#### 3.1.2. Surface Decoration with Bioactive Molecules

Functionalisation of BMSF-based scaffolds with bioactive agents represents a sophisticated approach to enhance cartilage regeneration by creating instructive microenvironments that support cell recruitment, proliferation, and differentiation. Prussian blue nanozyme nanoparticles incorporated into photocurable BMSF hydrogels yielded dose-dependent antioxidative and anti-inflammatory effects in vitro. Nanozyme concentrations ranging from 25 to 100 µg per 150 µL hydrogel significantly reduced intracellular ROS, preserved mitochondrial membrane potential, and suppressed Nod-like receptor protein-3 inflammasome activation. In vivo, the optimised nanozyme–BMSF hydrogel achieved complete cartilage resurfacing of rat femoral condyle defects by week 12, with no subchondral bone exposure, providing better outcomes than both BMSF-only and untreated control groups. These findings highlight that targeted oxidative stress can substantially enhance the cartilage repair capacity of BMSF-based scaffolds [131]. Similarly, melatonin with a final concentration of 2.77 mg/mL was incorporated into UV-crosslinked BMSF–gelatin methacrylate (GelMA) hydrogels, where melatonin acted as an intracellular antioxidant to preserve mitochondrial function. In vitro, melatonin-loaded BMSF–GelMA significantly upregulated chondrogenic markers relative to BMSF–GelMA alone. In a rabbit femoral condyle defect model, the melatonin–BMSF–GelMA constructs produced a continuous cartilage layer fully covering the defect and achieved higher ICRS scores at weeks 6 and 12 compared with both BMSF-only and untreated controls [132]. Tannic acid crosslinking into BMSF hydrogels exhibited comparable antioxidative properties with improved BMSC survival and ICRS scores [133]. Collectively, these findings suggest that antioxidant augmentation addresses the intrinsic lack of ROS modulatory capacity of BMSF. However, the long-term biosafety and degradation profiles of these antioxidant agents within joint microenvironments require further investigation.

Growth factor and differentiation agent incorporation represents another promising functionalisation strategy. BMSF also serves as an effective carrier for small-molecule chondrogenic inducers. For example, kartogenin was incorporated into BMSF–graphene oxide (GO) electrospun scaffolds with GO/Kartogenin mass ratios of 0.5, 1.5, and 3. Scaffolds with the highest GO/Kartinogen ratio (3:1) supported superior BMSC viability and matrix deposition in vitro, likely due to improved GO-mediated sustained release. When implanted subcutaneously in rats for 3 and 12 weeks, the high-ratio scaffolds exhibited greater ECM accumulation compared to low-ratio groups and non-functionalised BMSF controls. These findings suggest that GO-assisted molecular delivery can significantly enhance the bioactivity of BMSF scaffolds [134]. However, the relative contribution of kartogenin versus BMSF remains inadequately characterised due to a lack of appropriate control groups. Similarly, Xiao et al. observed significant defect recovery at 8 weeks using kartogenin-loaded freeze-dried silk scaffolds but lacked kartogenin-free controls to evaluate isolated effects [135]. More sophisticated approaches have employed TGF-β mimetic peptides (Ac-LIANAKGFEFEFKFK-NH_2_) into a photocurable BMSF–glycidyl methacrylate hydrogel scaffold, demonstrating excellent BMSC viability, proliferation, and chondrogenic differentiation in vitro. In vivo, the hydrogel enabled sufficient neo-cartilage deposition in a rabbit knee defect model within 3 months compared to BMSF only and no treatment controls [136]. Incorporation of platelet-rich plasma to BMSC-laden BMSF–GelMA hydrogels provided a growth factor reservoir. In vitro studies revealed that BMSC viability was dependent on GelMA concentration, with the highest tested amount (300 mg) exhibiting the most favourable cell survival. When injected into cartilage defects in rats, these BMSC-laden and platelet-rich plasma-containing hydrogels achieved significantly improved cartilage repair by 8 weeks, as shown by histological staining and lower Mankin scores compared to platelet-rich plasma-free hydrogels and untreated controls [137]. The study lacks evaluation of in vitro BMSC differentiation on hydrogels with an increased storage modulus resulting from high GelMA concentrations. Future work should include a comprehensive investigation of the mechanical properties of the modified BMSF biomaterials and their relationship to BMSC differentiation. Additional growth factors, including basic fibroblast growth factor [138] and combined bone morphogenetic protein-2 with TGF-β [65], have consistently enhanced chondrogenic differentiation compared to BMSF-only controls.

#### 3.1.3. Immunomodulation Approaches

The inflammatory microenvironment critically influences the fate of implanted biomaterials and the quality of cartilage regeneration, affecting the stability, proliferation, and migration of stem cells [139]. Whilst acute inflammation initiates chondrogenesis, prolonged immune responses direct BMSCs toward fibroblastic phenotypes that deposit collagen fibres, forming fibrocartilage rather than native hyaline cartilage [140]. Anti-inflammatory strategies have therefore become essential components of cartilage regeneration protocols. Synthetic corticosteroid methylprednisolone acetate (MPA) incorporation into BMSF–hyaluronic acid hydrogels demonstrated injectable feasibility beneficial for clinical translation [141]. Interestingly, MPA’s addition to BMSF–chitosan hydrogels did not significantly improve outcomes compared to BMSF-only groups, possibly due to BMSF’s inherent high biocompatibility being sufficient for inflammation minimisation [142].

**Figure 6:**
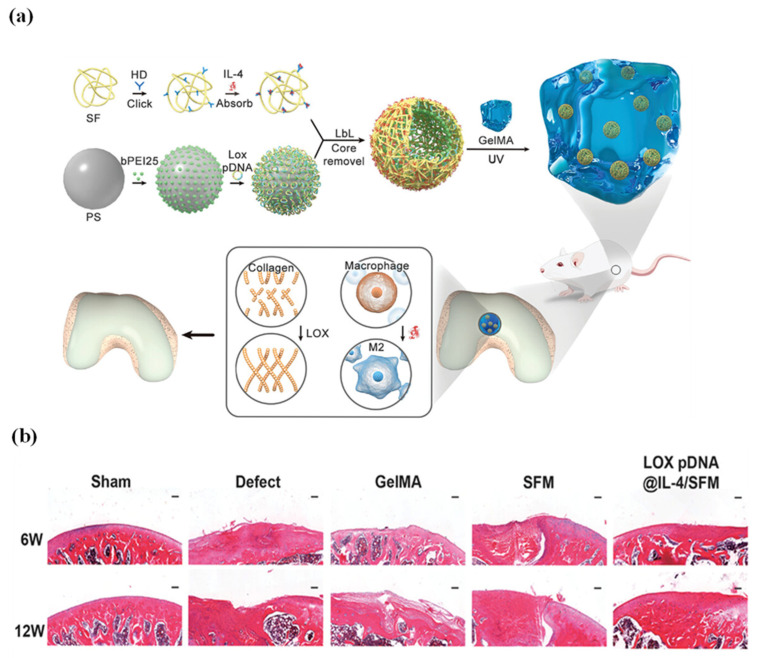
Haematoxylin and eosin staining post-hydrogel implantation. (**a**) Schematic diagram of the hydrogel preparation showing the rat femur cartilage defect model. (**b**) Representative images of the rat femur cartilage defect at 6 and 12 weeks with BMSF composite hydrogel (LOX pDNA@IL-4/SFM) compared to BMSF hydrogel only (SFM), hydrogel only (GelMA), and the control group. The results showed that the BMSF composite hydrogel group showed significant cartilage repair at 12 weeks after implantation. Scale bar = 100 μm. Reprinted with permission from Ref. [144]. Copyright 2023, John Wiley and Sons.

Alternative immunomodulatory approaches have targeted specific inflammatory pathways. TNF-α analogue etanercept prevents TNF-α receptor binding, demonstrating downregulation of catabolic factors such as matrix metalloproteinase-13 whilst facilitating collagen deposition in osteoarthritis chondrocytes and enhancing BMSC differentiation [143]. More sophisticated approaches have combined IL-4, an anti-inflammatory cytokine, with lysyl oxidase plasmid DNA loaded into BMSF microcapsules. To achieve a controlled release, BMSF microcapsules were encapsulated in GelMA hydrogels. In vitro results showed upregulating chondrogenic differentiation whilst promoting M2 macrophage polarisation [144]. In a rat knee cartilage defect model, only the group receiving the IL-4/lysyl oxidase-loaded BMSF microcapsule–GelMA hydrogel achieved smooth regeneration of hyaline cartilage lacking fibrotic tissue compared to BMSF only, GelMA only, and the negative control groups. This approach highlights the critical association between macrophage phenotype and chondrogenic outcomes, particularly in minimising fibrosis to restore normal joint functions (Figure 6). Multiple studies have consistently demonstrated that BMSF-based scaffolds combined with anti-inflammatory agents, including MPA, etanercept, or IL-4, lead to improved cartilage repair, as indicated by superior ICRS scores compared to untreated controls, emphasising the critical role of immune modulation in effective cartilage regeneration.

#### 3.1.4. Clinical Translation Considerations and Future Directions

Multiple studies have demonstrated that native BMSF alone is insufficient for thorough cartilage regeneration, requiring strategic modifications to facilitate the clinical translation of BMSF scaffolds in cartilage regenerative medicine. With the best optimal architecture and topographical features, BMSF scaffolds with cartilage origin bioactive molecules functionalisation is a significant approach to facilitate their clinical applications due to their inherent biocompatibility [145]. For instance, a BMSF–chondroitin sulfate composite scaffold significantly exceled pure BMSF in a rabbit osteochondral defect model, producing superior cartilage matrix deposition while reducing IL-1β-mediated inflammation and improved structural restoration after 6–12 weeks [146]. Similarly, a collagen-BMSF scaffold in a 7:3 ratio promoted robust chondrogenic differentiation in vitro and nearly complete hyaline cartilage recovery at 3–6 months in vivo [147]. These data suggest that functional biomimicry of native cartilage ECM is essential.

Moreover, a recent study showed that a BMSF scaffold engineered for sequential release of BMSF affinity peptide and TGF-β1 significantly improved cartilage regeneration in rabbits [148]. The results imply that synchronising the release of bioactive molecules with the recovering phase transition from soft to stiff can consolidate the clinical outcomes of BMSF by making them self-adaptive. However, the release kinetics need to be explored for safety.

A critical limitation across most of the studies is the lack of systematic comparisons with existing mature clinical treatments or widely used biomaterials. Therefore, future studies should prioritise comparative in vivo studies, standardised assessment protocols, and evaluation of scaffolds over extended time frames (>6–12 months).

### 3.2. Bone

Bone tissue has an inherent capacity for regeneration; however, large defects caused by trauma, tumour resection, or degenerative disease often exceed its natural healing potential and require clinical intervention. Current clinical options, such as autologous and allogeneic bone grafts, remain limited by donor site morbidity, restricted supply, and immune rejection risks [149,150]. These limitations have motivated intensive research into biomaterial-based strategies capable of achieving functional bone restoration. Among them, BMSF has gained attention for its mechanical robustness, tuneable degradation, and biocompatibility. Research has therefore focused on three major strategies: functionalising BMSF to enhance bioactivity, optimising its architecture to guide osteogenesis, and integrating angiogenic or neurogenic cues to promote tissue integration (see examples of BMSF-based biomaterials for bone regeneration in Table 5).

#### 3.2.1. Surface Decoration with Bioactive Molecules

**Figure 7:**
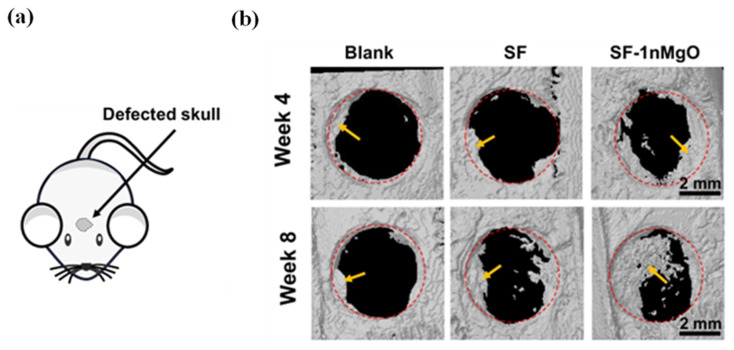
(**a**) Experimental design showing scaffolds were implanted into the cranial defects. (**b**) Micro-computed tomography images of the cranial defect sites were taken at weeks 4 and 8. Red circles outline the area of the defect, and yellow arrows point to new bone tissues. The BMSF and magnesium oxide composite group has the largest amount of new bone tissue inside the defect. Scale bar = 2 mm. Reprinted from Ref. [160] under the Creative Commons Attribution (CC BY 4.0) license. Copyright 2024.

Cranial defect repair remains one of the most challenging applications due to the complex geometry of skull defects [159]. A mechanically reinforced BMSF cryogel incorporating magnesium oxide nanoparticles showed great shape memory features for irregular shape bone defects. At different concentrations (0 wt%, 10 wt%, 30 wt%), the cryogel improved preosteoblast viability and promoted osteogenic differentiation of BMSCs in a dose-dependent manner, as indicated by upregulated osteocalcin and osteopontin expression. In vivo, a medium concentration (10 wt%, SF-1nMgO) of magnesium oxide nanoparticles had the lowest FBR as indicated in a subcutaneous model, which significantly led to increased bone volume after 8 weeks in the rat cranial defects compared with the BMSF-only group and untreated controls (Figure 7b) [160]. This study suggests that the concentration of functionalising bioactive molecules always requires optimisation to achieve optimal biocompatibility. However, mineralisation was not quantified, and the study lacked long-term evaluation—both critical factors for assessing repair of large defects. Extending beyond single-mineral modification, Zhu et al. combined amorphous calcium phosphate and platelet-rich plasma within oxidised BMSF hydrogels, designing a carboxyl-group-rich platform with oxidised BMSF for native cell seeding to promote bone regeneration. In the critical-size cranial defect model, after 12 weeks, these composites produced greater mineral deposition as represented by a higher volume of neo-bone in the defect sites than BMSF alone through activation of the PI3K/Akt pathway and increased mitochondrial calcium uptake [161]. It is worth noting that the increase in the carboxyl group of BMSF by oxidation successfully led to a greater bone regeneration area, indicating that the regenerative potential of BMSF can be improved by increasing binding sites for cells. In another study, lyophilised BMSF scaffolds coated with octacalcium phosphate and amorphous calcium phosphate, which can be referred to as biomimetic calcium phosphate coatings, were loaded with TGF-β, bone morphogenetic protein 2. The coating serves as a protective layer that allows for a controlled release of proteins. Dual protein incorporation resulted in better synergistic enhancement of trabecular formation and bone volume in a femur osteochondral defect model at weeks 5 and 10 than the no coating control and BMSF-only groups, suggesting the significance of bioactive molecules’ control release [162]. While these studies demonstrate improved osteoinduction, they provide limited data on release kinetics, long-term performance, and scaffold–tissue interactions, which are factors essential for translation. These challenges have encouraged exploration of cell-based functionalisation approaches.

Functionalising BMSF with living stem cells has emerged as an alternative to biochemical modification. Incorporating BMSCs as 3D spheroids within BMSF matrices improved osteogenic differentiation compared with BMSCs alone, attributed to enhanced cell–cell communication and the intrinsic cytocompatibility of BMSF [163]. This study implies that BMSF-based spheroids may serve not only as improved cell carriers, but also as micro-architectures that better control spatial organisation and differentiation cues in bone regeneration therapies. Despite promising outcomes, autologous cell use presents challenges for clinical scalability due to cost, cell viability maintenance, and the potential malignant transformation of BMSCs. Consequently, attention has shifted towards architectural optimisation of acellular scaffolds.

#### 3.2.2. Impact of *Bombyx mori* Silk Fibroin Scaffold Architecture on Bone Regeneration

Architectural control profoundly influences osteogenesis. Ai et al. demonstrated that small-pore BMSF scaffolds (160–220 μm) coated with gelatine and fibronectin promoted higher osteogenic gene expression in BMSCs than medium (250–315 μm) or large-pore (350–400 μm) scaffolds, although in vivo validation and precise porosity quantification were lacking [164]. The small-pore-size group exhibited the greatest surface area relative to the other scaffold groups, resulting in higher adsorption of fibronectin and gelatine proteins. This implies that decreasing pore size increases the density of available adhesion sites for integrin binding; therefore, enhancing the initial protein conditioning of the BMSF surface is an essential determinant of subsequent cell attachment, spreading, and mechanotransduction. Contrasting findings have also been reported. Scaffolds with lower porosity exhibited greater osteogenic marker expression [165], while those engineered with porosity gradients showed enhanced cell viability and bone deposition with high porosity BMSF scaffolds from week 4–8 in knee joint defect models by facilitating osteoblast infiltration and proliferation [166]. These divergent results suggest that porosity and pore size act synergistically rather than independently. Indeed, high porosity combined with small pore size (191.6 ± 3.7 μm) upregulated osteoblast-related gene expression [167], highlighting the importance of local geometry. Mechanical stiffness further modulates osteogenic behaviour: Yang et al. fabricated BMSF–polyethylene glycerol composite scaffolds with various stiffness by adjusting the amount of hydroxyapatite, ranging from 80 to 150 kPa. Results showed scaffolds with 120 kPa demonstrated the highest level of osteogenic differentiation in vitro, and the majority of the defect area in the cranial model was filled by neo-bone thick layers [168]. A stiffness of approximately 120 kPa most effectively activates integrin-dependent mechanosensitive signalling, likely by promoting stable focal adhesion assembly and adequate cytoskeletal tension. Matrices that are either too soft or stiff fail to support this balance, thereby interrupting mechanotransduction and reducing chondrogenic responsiveness. Collectively, these findings indicate that architecture and stiffness jointly regulate osteogenesis through mechanoresponsive pathways. However, while architectural optimisation supports osteogenic differentiation, it alone cannot ensure vascular and neural integration, both of which are critical for sustaining bone tissue function.

#### 3.2.3. Vascularisation and Neurogenesis Are Essential for Bone Regeneration

Vascularisation is essential for nutrient transport and osteoblast function during bone formation [169,170]. Kim et al. developed a two-phase system using angiopoietin-1-functionalised BMSF/Broussonetia kazinoki scaffolds, which were pre-vascularised before implantation with stromal cell-derived factor-1 treatment. This approach significantly increased bone volume compared with unvascularised and no stromal cell-derived factor-1 controls [171]. To simplify such multi-step methods, Deng et al. encapsulated melatonin within BMSF electrospun scaffolds, achieving enhanced angiogenesis via the PI3K/Akt pathway and increased mineralisation at weeks 4 and 8 in the cranial defects relative to the negative control and BMSF-only scaffolds [172]. Likewise, BMSF scaffolds supporting human amniotic mesenchymal stem cells promoted both osteogenic and angiogenic differentiation, successfully promoting better healing outcomes in critical-sized bone defects at week 8 [173]. Although promising, these results require validation in large-animal models to evaluate immune compatibility and long-term functionality. Collectively, these studies particularly emphasise the importance of nutrient transport in bone healing via the vasculature, highlighting that further studies should focus on the mechanical and architectural properties of BMSF scaffolds in favour of vascularisation.

Neurogenesis also contributes to bone repair, as neural and osteogenic signalling pathways are interlinked through Erk1/2-mediated mechanisms [174]. To exploit this relationship, Wang et al. engineered electrospun biomimetic BMSF scaffolds incorporating nerve growth factor, osteogenic proteins (osteocalcin, osteopontin, biglycan), magnesium bioactive glass, and PCL. These scaffolds induced neural differentiation of BMSCs in vitro, resulting in higher expression of osteogenic markers compared with scaffolds lacking nerve-growth-factor functionalisation. In a rat cranial defect model, the nerve growth factor-loaded BMSF scaffolds promoted significantly greater bone regeneration by week 4, accompanied by enhanced neurogenesis within the newly formed Haversian canals [175]. This study provides direct evidence that neural signalling contributes to osteogenesis through Erk1/2 downstream pathways, highlighting a previously underexplored strategy in which neurogenic functionalisation of BMSF scaffolds may be leveraged to accelerate and improve bone regeneration. Despite their effectiveness, the complexity of such formulations raises challenges for reproducibility, biosafety, and cost control.

#### 3.2.4. Clinical Translation Considerations and Further Directions

Despite extensive progress in BMSF biomaterials for bone regeneration, several challenges still hinder the clinical translation of BMSF bone scaffolds. A major challenge is achieving mechanical compatibility between BMSF scaffolds and the native bone microenvironment. Although BMSF possesses favourable intrinsic strength, unmodified scaffolds often fall short of matching the compressive strength of native bone. Compositing BMSF with calcium phosphates has shown promise in providing the required stiffness to withstand mechanical loading while simultaneously enhancing the osteoconductivity of the scaffold [152]. The host immune response to serine remnants in BMSF scaffolds also requires further optimisation. Recent studies suggest that integrating carbonate hydroxyapatite into BMSF scaffolds can promote pro-osteogenic M2 macrophage polarisation via the JAK/STAT signalling pathway, thereby enhancing in vivo bone formation while simultaneously providing mechanical properties that better match native tissue [176]. The unique features of the bone microenvironment restrict the design of BMSF biomaterials. Therefore, future research should focus on tuning scaffold architecture and degradation profiles to more closely align with the kinetics of bone healing.

### 3.3. Skin

The skin, the body’s largest organ, acts as a critical barrier against mechanical, chemical, and microbial insults while regulating water balance. Severe burns, chronic wounds, and surgical defects can overwhelm its regenerative capacity, leading to delayed healing, infection, and scarring [177]. Conventional interventions like autografts, allografts, and synthetic dressings offer temporary coverage but suffer from donor site morbidity, immune rejection, and poor integration [178]. Consequently, there is a growing need for biomaterials that not only protect the wound but also actively orchestrate tissue repair. Recent studies based on BMSF-based constructs for skin regeneration can be grouped into four major strategies: exploiting its intrinsic cytocompatibility, enhancing its bioactivity through biochemical functionalisation, tailoring its architecture, and modulating immune responses to promote regenerative healing. Examples of recent BMSF-based approaches for skin regeneration are summarised in Table 6.

#### 3.3.1. *Bombyx mori* Silk Fibroin Is a Promising Biomaterial for Skin Healing

**Figure 8:**
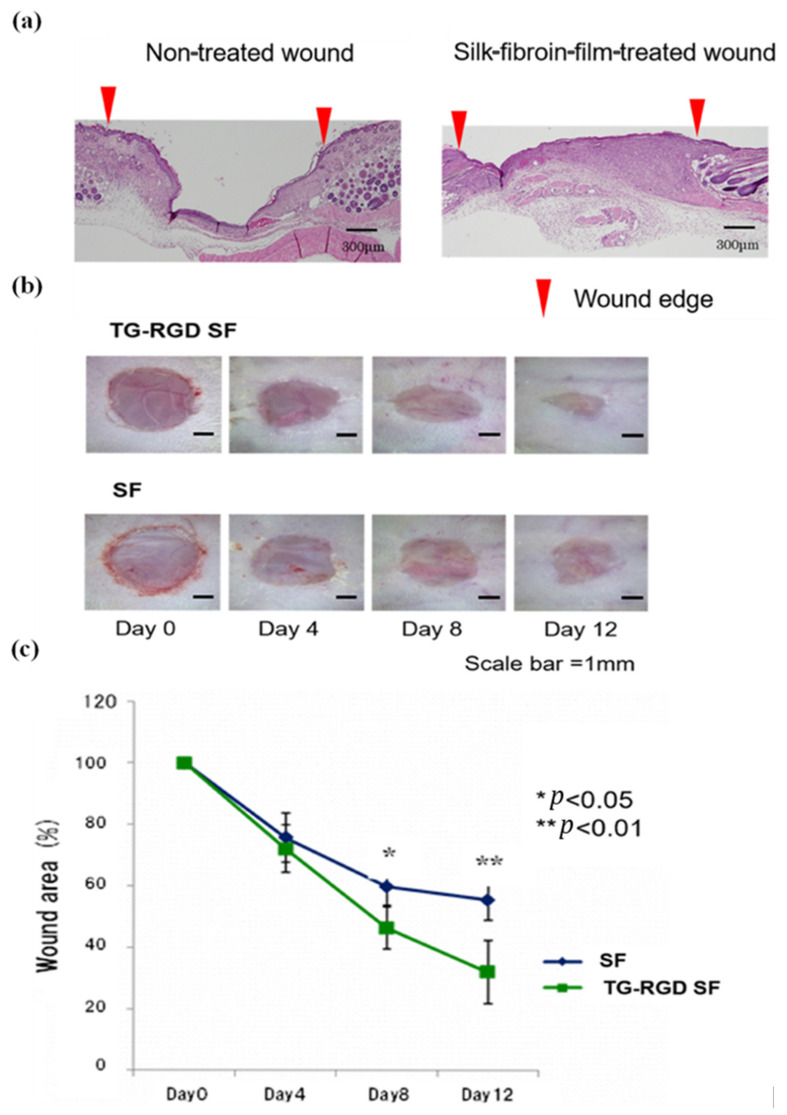
Skin wound healing. (**a**) Immunohistostaining of the wound on day 12 post-injury. The BMSF-treated group shows a thicker dermis compared to the no-treatment control, suggesting better healing outcomes. Scale bar = 300 μm (**b**). Representative images of wound closure from days 0–12. Scale bar = 1 mm. (**c**) The transgenic RGD silk group had a faster wound closure rate than the standard BMSF group. Reprinted with permission from Ref. [190]. Copyright 2024, John Wiley and Sons.

Application of BMSF hydrogel to full-thickness murine skin defects led to a faster rate of closure relative to chemically modified hydrogels containing tannic acid or acrylamide-dopamine, with clear differences evident by day 12. Parallel antibacterial assays demonstrated that native BMSF exhibits robust intrinsic antibacterial activity comparable to that of the chemically enhanced variants. This indicates that the unmodified BMSF matrix already provides a favourable healing environment by limiting bacterial proliferation and reducing early infection-associated inflammation [187]. Similarly, a stretchable BMSF film accelerated wound closure over 21 days relative to untreated controls, demonstrating the therapeutic efficacy of native BMSF [188]. Hashimoto et al. compared BMSF sponges with collagen scaffolds and reported greater keratinocyte migration with more lamellipodia, upregulation of differentiation-related genes, and downregulation of fibrosis-associated genes, translating to reduced scarring [189]. The intrinsic biocompatibility of BMSF is sufficient to promote cell migration by enabling a higher level of spreading compared to collagen scaffolds. A direct comparison between the BMSF group and the tissue culture plastic control group is needed to evaluate the absolute performance of BMSF. Despite these benefits, native BMSF lacks the RGD motif essential for integrin-mediated adhesion. Transgenic BMSF incorporating RGD sequences achieved faster wound closure and reduced granulation tissue formation compared with unmodified films at day 12 (Figure 8) [190]. The presence of the RGD sequence can lead to a more enhanced response of integrin-mediated pathways, particularly MAPK pathways that promote endothelial cell adhesion, thereby promoting angiogenesis, subsequently resulting in a better healing outcome compared to BMSF. Collectively, these findings confirm that while BMSF is intrinsically cytocompatible, its regenerative potential can be further optimised through molecular design.

#### 3.3.2. Functional Modification of BMSF Scaffolds with Bioactive Molecules

To enhance cell adhesion and control tissue responses, BMSF has been combined with natural polymers and bioactive agents. A composite of BMSF, hyaluronic acid, and natural silk fibres produced scar-suppressive effects in a rabbit ear model by downregulating collagen-I, TGF-β, and α-smooth muscle actin expression [191]. The natural silk fibres in the outer layer effectively maintained a moist wound environment, whereas the hyaluronic-acid–BMSF porous inner layer increased cell adhesion sites and facilitated deeper cellular infiltration. This configuration worked synergistically to recreate key features of native skin architecture, resulting in improved healing outcomes. In another strategy, magnesium-chloride-incorporated BMSF films significantly improved epidermal regeneration at day 20, as magnesium and BMSF synergically attenuated oxidative stress and inflammation and promoted angiogenesis, collagen deposition, and epidermal regeneration. The results highlight that oxidative stress and inflammation are key factors hindering wound closure [192]. A more complex approach by Khosrowpour et al. used extracellular matrix (ECM) harvested from decellularised placenta blended with BMSF as bioink for 3D printing. The resulting constructs accelerated closure and enhanced expression of TGF-β, collagen, and vascular endothelial growth factor (VEGF) genes across multiple time points [193]. Interestingly, the BMSF-only control also significantly improved healing, underscoring its intrinsic bioactivity. Despite these advances, few studies have systematically examined underlying signalling pathways, particularly those governing macrophage behaviour, a central regulator of inflammation and tissue remodelling. Understanding these mechanisms is critical for developing immunologically informed BMSF designs.

#### 3.3.3. Immune Modulation and Macrophage Polarisation

Delayed healing, particularly in diabetic wounds, is strongly associated with prolonged M1-type macrophage activation and inadequate transition to the reparative M2 phenotype [194,195,196]. Several recent studies have therefore explored how BMSF can modulate macrophage polarisation. Lactobacillus rhamnosus biofilm-coated BMSF/polycaprolactone (PCL) electrospun films displayed potent antibacterial activity and induced M2 polarisation, as shown by increased CD206 and reduced IL-6 expression, correlating with accelerated wound closure [197]. Similarly, silver-ion-loaded BMSF hydrogels enhanced healing by limiting bacterial growth and inflammation [198]. Although the long-term cytotoxicity and persistence of silver remains a concern, crosslinking an endothelial growth factor-mimetic peptide KLTWQELYQLKYKGI (QK) into BMSF hydrogels further stimulated vascularisation and M2 macrophage polarisation, yielding denser collagen deposition and improved dermal organisation [199]. These results highlight the immunomodulatory potential of BMSF scaffolds, and regulating the polarisation of macrophages is the key to accelerating wound closure. However, questions remain about the safety, metabolic stability, and relative efficacy of incorporated bioactive molecules. Moving forward, an understanding of how scaffold structure alone modulates immune behaviour may reduce dependence on exogenous agents.

#### 3.3.4. Architectural Modulation of Immune Response

The topography and porosity of BMSF surfaces can actively influence macrophage phenotype and healing kinetics. Yang et al. fabricated two types of BMSF films with distinct groove dimensions. In vitro results demonstrated that BMSF films patterned with larger microgrooves (400 μm in width, 400 μm apart, 200 μm in depth) promoted M2 polarisation accompanied by increased expression of collagen, TGF-β, and fibronectin compared with BMSF films with smaller microgrooves (100 μm in width, 50 μm apart, 25 μm in depth). The larger-groove BMSF films enhanced collagen deposition and vascularisation, leading to significantly faster wound closure between days 7 and 14 compared with smooth films. These findings highlight that topographical cues can be strategically engineered to modulate immune responses and tissue regeneration by regulating gene expression through mechanosensitive signalling pathways that regulate macrophage polarisation [102]. Porous BMSF films have shown comparable benefits: Liu et al. reported enhanced cell migration in vitro and faster wound contraction in vivo relative to non-porous controls [200,201]. Porosity likely facilitates cell infiltration, vascularisation, and moisture exchange; however, most studies lack a quantitative correlation between pore size, stiffness, and immune behaviour. In particular, the combined influence of pore geometry and mechanical compliance on macrophage polarisation remains underexplored, despite its significance in establishing a pro-regenerative microenvironment.

#### 3.3.5. Clinical Translation Considerations and Further Directions

Multiple strategies have been employed to engineer BMSF-based biomaterials for wound healing, and although these systems have demonstrated promising outcomes, several challenges still hinder their clinical translation. One major limitation is that BMSF degradation must be precisely matched to the kinetics of skin repair [202]. The degradation profile of BMSF heavily depends on the processing techniques and the resulting formats, a mismatch that can lead to hindered epithelial regeneration [203]. Optimising the architecture of BMSF biomaterials for skin healing is needed. In addition, although some evidence has shown that BMSF with bioactive molecules can promote angiogenesis while minimising scarring in wound closure, it remains a major conflict in skin healing. Over angiogenesis is associated with active fibroblast scar formation [204]. Further research should focus on designing composite architectures and bioactive molecules to achieve angiogenesis and scar tissue modulation simultaneously.

### 3.4. Nervous Tissue

Neural injuries involving both the central and peripheral nervous systems remain major clinical challenges due to the limited regenerative capacity of neural tissues. In the central nervous system (CNS), regeneration is hindered by the formation of a glial scar—a dense inhibitory structure composed of reactive astrocytes and extracellular matrix (ECM) molecules that obstruct axonal regrowth [205]. In contrast, effective debris clearance is critical for successful regeneration in the peripheral nervous system (PNS) [206]. Current treatment options, including autografts and nerve conduits, are constrained by donor site morbidity and limited bioactivity. These shortcomings highlight the need for biomaterials capable of promoting complex nerve repair. In this context, BMSF has shown encouraging results (see examples in Table 7). Recent strategies can be broadly categorised into three groups: incorporation of bioactive molecules, cell-based approaches, and immunomodulation.

#### 3.4.1. Central Nervous System

Multiple studies have demonstrated the potential of BMSF-based systems to promote CNS repair through biochemical or cellular integration. The use of neural stem cells (NSCs) is among the most direct strategies for neuronal regeneration. For example, 3D-printed BMSF–collagen composite scaffolds with a stiffness of (60.05 ± 5.12 kPa) matched the stiffness of spinal-cord-supported NSC culture and, in vivo, enabled both functional and histological recovery. In these models, NSC-seeded 3D BMSF constructs exhibited the highest axonal density, the fastest motor function recovery, and significantly reduced glial scar formation compared with acellular scaffolds and the negative controls [214]. Similarly, in hypoxic-ischemic encephalopathy rats, NSCs delivered within aligned BMSF hydrogels significantly accelerated long-term spatial learning and cognitive recovery without further enhancing neuronal regeneration or motor function. This improvement correlated with early and sustained neurotrophin secretion and increased synaptic protein expression, highlighting the hydrogel’s ability to preserve NSC paracrine activity [215]. These findings confirm that BMSF supports NSC viability, although long-term cell survival, differentiation, and safety, particularly in terms of tumorigenic risks, remain to be addressed.

To overcome the complexity of stem-cell-based approaches, functionalisation with bioactive molecules has emerged as a more translationally feasible strategy. For example, 3D-printed collagen/silk fibroin scaffolds loaded with the secretome of human umbilical-derived mesenchymal stem cells significantly improved functional recovery in a rat model of spinal cord injury. When compared with scaffolds alone, secretome-loaded constructs enhanced hindlimb locomotor outcomes while also promoting greater axonal regeneration, remyelination, and synaptic connectivity at the injury site. This evidence highlights that the incorporation of MSC secretome can stabilise and potentiate neuroregenerative processes within BMSF-based scaffolds, offering a safer alternative to direct cell transplantation [216]. Chen et al. incorporated oxidised dopamine into injectable BMSF hydrogels, finding that at the optimal dopamine concentration, the hydrogel increased axonal length and neuron proliferation in vitro and enhanced neuronal density in vivo compared to untreated controls within two weeks post-implantation in a rodent spinal cord hemisection model [217]. This study also suggests that, in addition to the bioactivity of dopamine, the modulation of hydrogel porosity and stiffness resulting from dopamine incorporation is crucial for determining the regenerative capacity of BMSF hydrogels. Similarly, BMSF hydrogels combining brain ECM and calcium peroxide mitigated hypoxia-induced cell death by modulating oxygen release while maintaining CNS-compatible stiffness; however, the lack of in vivo validation limited translational relevance [218]. Despite promising short-term outcomes, most studies overlook long-term integration and inflammatory regulation, which are pivotal for sustained neural recovery. Microglia–astrocyte interactions are key drivers of glial scar formation [219], emphasising the importance of anti-inflammatory strategies.

**Figure 9:**
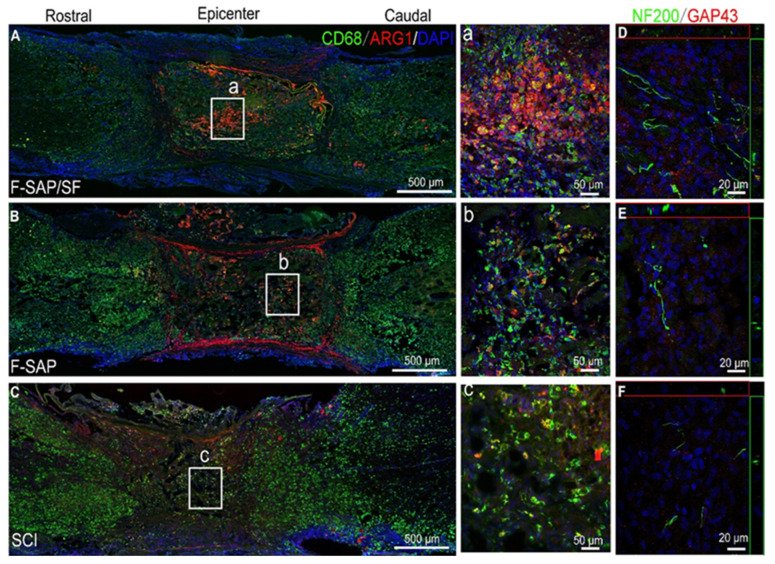
Immune and neuron regeneration characterisation at the SCI implantation site of the hydrogel. (**A**–**C**): Representative images of M2 macrophage staining, the pan macrophage marker CD-68 (green), and the M2 anti-inflammatory macrophage marker ARG-1 (red). (**a**–**c**): Zoomed-in images of the implantation site. (**D**–**F**): Representative images of newly generated neurons indicated by colocalisation of NF200 (green) and GAP43 (red). Reprinted with permission from Ref. [221]. Copyright 2024, The American Association for the Advancement of Science.

Anti-inflammatory exosome-loaded BMSF hydrogels demonstrated dual benefits of sustained exosome release and immune modulation, leading to improved axonal regeneration and motor recovery in spinal cord injury models [220]. This enables spinal cord injury mice to achieve 80% of sham motor function recovery at 28 days post-injury, providing a novel injectable, biosafe, multi-target synergistic biomaterial solution for clinical translation. Likewise, Feng et al. combined BMSF with functional self-assembling peptides mimicking the native ECM of nervous tissue, which promoted M2 macrophage polarisation, reduced pro-inflammatory cytokines, and enhanced myelination in the corticospinal tract [221]. Immunostaining revealed increased M2 macrophage marker-Arginase-1 expression and co-localisation of neurofilament-200 with growth-associated protein-43, indicating that neuronal maturation and sprouting are closely linked to M2 macrophage activity (Figure 9). This work demonstrates the integration of ECM simulation and immune modulation into a single hydrogel, achieving functional reconstruction. This simple formulation strategy provides a viable matrix platform for spinal cord injury treatment. Qi et al. further developed a BMSF–hyaluronic acid–dopamine platform enabling sustained delivery of Neurotrophin-3, which enhanced axonal regeneration and upregulated anti-inflammatory cytokines while suppressing pro-inflammatory ones [222]. Similarly, BMSF hydrogels functionalised with basic fibroblast growth factor promoted neurogenic differentiation, induced mature neuron marker microtubule-associated protein-2 expression, and favoured M2 macrophage polarisation both in vitro and in vivo [223]. Taken together, these studies highlight that BMSF-based scaffolds can be functionalised with growth factors or neurotrophic agents to synergistically enhance neuronal regeneration and modulate post-injury inflammation, particularly macrophage polarisation, demonstrating that immune regulation is a key future strategy in regenerative medicine with BMSF biomaterials.

#### 3.4.2. Peripheral Nervous System

Comparable approaches have been explored for peripheral nerve repair. BMSF scaffolds pre-seeded with stem cells facilitated axonal regeneration [224,225] by providing a biocompatible and supportive matrix that guides neurite outgrowth, whereas decoration with bioactive molecules offered higher translational potential. Electrospun BMSF–metformin scaffolds fabricated on chitosan substrates supported oriented Schwann cell alignment and dorsal root ganglion axon extension in vitro [226,227]. Gene expression analysis confirmed upregulation of migration and proliferation-related genes [226], suggesting that the bioactive scaffold not only provides topographical guidance but also activates intrinsic regenerative pathways. However, the absence of in vivo studies limits clinical interpretation.

Gao et al. designed composite BMSF–graphene oxide hydrogels incorporating fibroblast-derived exosomes fabricated via a three-step click chemistry approach, which enhanced both electrical conductivity and exosome-mediated signalling. In vitro, this system promoted axon elongation and myelination, indicating that the conductive, bioactive scaffold supports neuronal maturation. In vivo, implantation facilitated functional recovery of the sciatic nerve and promoted concomitant vascularisation via activation of the NOTCH signalling pathway, which correlated with enhanced neural repair and tissue integration [228]. Similarly, Xiang et al. developed vascular endothelial growth factor-loaded BMSF composite conduits that enhanced endothelial cell and Schwann cell proliferation in vitro [229], though in vivo validation is still required. Overall, these studies underscore the role of vascularisation in BMSF-mediated peripheral nerve regeneration and the potential of biofunctionalised scaffolds to integrate angiogenic and neurogenic cues for nerve repair.

Inflammation plays a decisive role in peripheral nerve regeneration, as macrophage polarisation toward the M2 phenotype supports axonal repair [230,231]. BMSF hydrogels crosslinked with zinc nanoparticles carrying axonal growth-promoting miRNA induced neurogenic differentiation of PC12 cells and suppressed inflammatory mediators (TNF-α, IL-6, NOS2) in macrophages [232]. Even unmodified BMSF exhibited intrinsic immunoregulatory effects: freeze-dried tubular BMSF grafts implanted into sciatic nerve defects restored motor function, reduced muscle atrophy, and increased myelination compared with silicone tube controls. Immunostaining confirmed higher M2 macrophage density in the BMSF group relative to sham and autologous grafts [233]. The intrinsic biocompatibility of BMSF contributes to M2 macrophage polarisation, suggesting that BMSF serves as a versatile platform for further modifications to enhance neural healing outcomes.

#### 3.4.3. Clinical Translation Considerations and Further Directions

BMSF has increasingly emerged as a key biomaterial in neural tissue engineering due to its excellent biocompatibility and tuneable mechanical properties. Despite these advantages, a major limitation of BMSF is its lack of intrinsic electrical conductivity, which is critical for neuronal stimulation [234]. Composite strategies incorporating conductive polymers or nanoparticles have been explored to provide electrical cues [235]. However, balancing conductivity and cytocompatibility remains challenging. Therefore, future BMSF biomaterials for nerve regeneration should integrate electrical conductivity, pro-neurogenic bioactive molecules, and spatially aligned microtopography to closely mimic the native extracellular matrix, thereby guiding and enhancing neural regeneration.

### 3.5. Vasculature

Autologous grafts remain the gold standard for vascular regeneration owing to their reduced rate of immune rejection. However, their clinical use is often restricted by patients’ comorbidities. These constraints have driven research interest in vascular biomaterials. Currently available biomaterials for vascular regeneration primarily consist of synthetic polymers, including ePTFE, commercially known as Gore-Tex^®^, and polyethylene terephthalate. While these materials demonstrate adequate performance in large-diameter vascular grafts (>6 mm) with satisfactory long-term patency rates, their inherent limitations, including excessive stiffness, chemical inertness, and hydrophobicity, significantly compromise biocompatibility [236]. In large-diameter grafting, they perform great with a long survival rate; however, in small-diameter grafting (<6 mm), they often fail with failure rates of 75% [78,237]. The predominant failure mechanism involves inflammation-induced neointimal hyperplasia (NIH) characterised by pathological vascular smooth muscle cell (VSMC) proliferation as a result of inadequate endothelialisation. Thus, there is an urgent need to discover a new biomaterial for vascular regeneration. BMSF has emerged as a promising biomaterial in revascularisation owing to its biocompatibility with endothelial cells and hemocompatibility, which is unique to synthetic polymers. Examples of recent BMSF-based approaches for vascular regeneration are summarised in Table 8. Recent strategies are central to promoting endothelialisation to minimise NIH. They can be broadly categorised into functionalising BMSF vascular grafts with bioactive molecules, optimising the architecture of BMSF vascular grafts, and immunomodulation.

#### 3.5.1. Surface Decoration with Bioactive Molecules

Functionalisation of BMSF vascular grafts with bioactive molecules has emerged as a key strategy to enhance endothelialisation and modulate cellular responses. In particular, BMSF functionalised with the Arg–Glu–Asp–Val motif peptide provides an integrin binding site to make up for the absence of the RGD sequence in mulberry silk. In vitro, endothelial cells demonstrated superior proliferation and spreading on modified BMSF films while inhibiting smooth muscle proliferation [244,245]. These findings indicate that the restoration of integrin-mediated signalling can modulate the balance between endothelialisation and NIH, improving the regenerative performance of BMSF vascular grafts. More recently, electrospun scaffolds composed of BMSF blended with collagen I and polyglycerol sebacate were further functionalised with syndecan-4 and stromal-cell-derived factor-1α to enhance endothelial cell recruitment and retention. Endothelial colony-forming cells showed significantly greater spreading on the functionalised scaffolds, and qPCR data confirmed higher expression of endothelial markers CD31 and von Willebrand factor [246]. Together, these results indicate that integrating chemotactic cues with adhesion-modulating molecules can synergistically improve endothelialisation within BMSF-based vascular constructs. Similarly, a study coaxially electrospun BMSF with vascular endothelial growth factor and TGF-β inhibitors for sequential release. They found enhanced endothelialisation while preventing over-deposition of collagen fibres in vitro [247]. These dual-delivery systems introduce a more sophisticated level of bioactivity modulation, raising concerns regarding reproducibility, release kinetics in vivo, and potential off-target effects. Overall, these studies converge on a shared design principle: accelerating and stabilising endothelialisation on the surface of grafts is the most effective strategy for preventing NIH, and BMSF-based vascular grafts provide a highly supportive platform for achieving this through diverse biochemical and structural modifications. The above studies lack immune characterisation, which is a direct factor contributing to the development of NIH.

BMSF coated with polyurethane, which is capable of releasing nitric oxide, was also effective in preventing the proliferation of VSMCs and enhancing endothelial cell spreading and proliferation while modulating the immune environment. Macrophage response flow cytometry assay showed macrophage polarisation was directed to M2, while enzyme-linked immunosorbent assays showed a higher level of anti-inflammatory cytokines in vitro [248]. However, the in vivo nitric oxide release profile was not characterised, and the interplay between nitric oxide delivery and long-term matrix degradation remains insufficiently understood, limiting the strength of its clinical translation. Gupta et al. conducted a comprehensive study on BMSF vascular grafts functionalised with Wharton’s Jelly, showing a higher M2/M1 ratio than the BMSF-only group, exhibited 100% patency after 2 months of jugular implantation in rabbits and a higher degree of endothelialisation [249]. Although these studies have demonstrated strong potential for clinical translation, they all lack blood flow simulation in vitro. Evaluating endothelialisation under static conditions, therefore, provides an incomplete understanding of scaffold performance. Endothelial cells in normal vasculature are exposed to vascular physiological factors: shear stress derived from blood flow and blood pressure. As previously discussed, cells are mechanosensitive, and the biology of endothelial cells depends on mechanosensation to these factors, such as spreading, expression of adhesion molecules, and alignment [88,250]. Given the mechanosensitive nature of endothelial cells, evaluating endothelialisation in static in vitro environments provides an incomplete understanding of scaffold performance. To address this gap, bioreactor systems that simulate physiological flow conditions are increasingly recognised as necessary tools for preclinical scaffold assessment [88]. In addition to the previous studies, Jiang et al. functionalised BMSF with perlecan domain V, which serves as a binding site for integrins, and showed enhanced endothelial cell spreading and proliferation, with less proliferation of smooth muscle cells under physiological flow [251].

While this represents an important advancement by incorporating shear stress into scaffold evaluation, it remains an exception rather than the norm. Broader adoption of flow-based models is essential to bridge the gap between in vitro findings and in vivo vascular performance. In addition, surface architecture is a crucial but also fundamental factor in determining the adhesion of endothelial cells and macrophage polarisation [83,88]. Therefore, a strong focus on manipulating the architecture of the BMSF vascular graft, aiming to improve the survival rate, is required.

#### 3.5.2. Impact of the Microarchitecture and Mechanical Properties of BMSF Vascular Grafts on Their Performance

**Figure 10:**
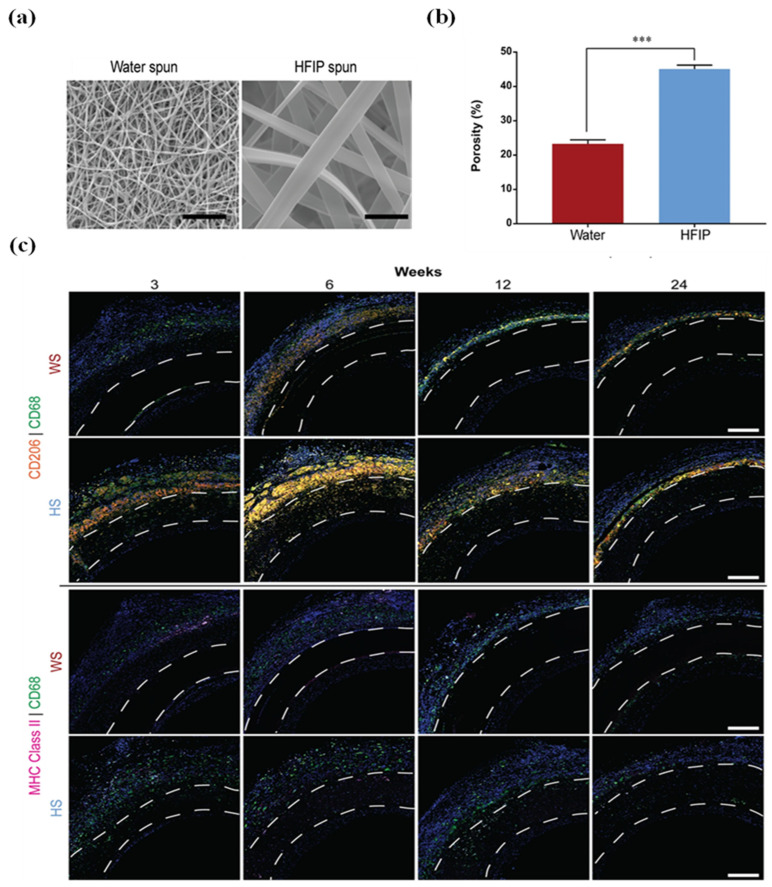
(**a**) Scanning electron microscopy images of HFIP and water electrospun BMSF scaffolds. Scale bar = 10 μm. (**b**) HFIP electrospun scaffolds had significantly higher porosity than water. *** *p* < 0.001. (**c**) Representative images of immunofluorescence staining of macrophages in BMSF vascular grafts after rat aortic transplantation at weeks 3, 6, 12 and 24. The pan macrophage maker CD68 (Green), M1 marker MHC class II (Purple) and M2 marker CD206 (Orange). Results show that higher porosity HFIP electrospun (HS) induced a higher level of CD206 at week 6 compared to water electrospun vascular grafts (WS) with lower porosity. Scale bar = 200 μm. Reproduced from [Chan et al., Scientific Reports, 2019], Nature, licensed under CC BY 4.0. Reprinted from Ref. [253]. under the Creative Commons Attribution (CC BY 4.0) license. Copyright 2019.

Electrospinning is the most common fabrication technique for synthetic vascular grafts, enabling tuneable control over scaffold architecture. Filipe et al. demonstrated that BMSF electrospun scaffolds support superior endothelial cell spreading and survival compared with ePTFE scaffolds after 24 weeks of rat aortic implantation [81]. Despite the head-to-head comparison emphasising the performance of BMSF in revascularisation, scaffold porosity, an inherent feature of electrospun scaffolds, lacked quantification. A more recent study has highlighted that adjusting the solvent used for BMSF dissolution can significantly impact the porosity and biological performance of BMSF vascular grafts [252]. By dissolving BMSF in HFIP, compared to waterspun silk vascular grafts, HFIP electrospun grafts resulted in higher porosity, leading to thinner NIH with more M2 and fewer M1 macrophages post-implantation (Figure 10) [252,253]. Results also showed higher endothelial coverage in HFIP-spun silk vascular grafts, suggesting enhanced hemocompatibility and integration. However, these studies lack a deep analysis of the mechanism of BMSF scaffold stiffness, which in the case of electrospinning, is proportional to porosity. Macrophages sense matrix stiffness through mechanosensors such as Piezo1/2, leading to cytoskeletal remodelling and integrin-dependent M1 polarisation via nuclear YAP translocation and the HIPPO pathway [254,255,256]. Therefore, the architecture and mechanical properties of BMSF can regulate immune responses to prolong the survival of small-diameter BMSF vascular grafts [83]. In general, although results are promising, the translation of these strategies requires further investigation into the interplay between porosity, mechanical features, and host immune response.

#### 3.5.3. Clinical Translation Considerations and Further Directions

BMSF has significantly better biocompatibility than synthetic polymers, making it have a higher survival rate in small-diameter applications. Despite the promising performance of BMSF, several BMSF-specific challenges remain that are critical to address for clinical translation. BMSF has higher intrinsic stiffness than native blood vessels, which significantly impairs compliance and leads to flow mismatch, causing interrupted endothelialisation under dynamic conditions [257]. Future research should primarily focus on precisely tuning the scaffold architecture and surface topography to modulate macrophage polarisation, as inflammation mediated by macrophages directly contributes to NIH. Subsequently, BMSF-based vascular grafts should be evaluated in large animal models to better predict clinical performance and long-term outcomes.

### 3.6. Cornea

Corneal injuries and corneal diseases are the leading cause of vision impairment worldwide and often require surgical interventions such as corneal transplantation. However, the shortage of donor tissue, the risk of immune rejection, and the limited integration of synthetic implants pose significant clinical challenges [258]. Tissue engineering offers promising alternatives, and BMSF has emerged as a versatile biomaterial for corneal regeneration [259,260]. BMSF has excellent optical clarity, mechanical strength, biocompatibility, and tuneable degradability, making it ideally suited for replicating the native corneal microenvironment [261]. See examples in Table 9. Recent studies involving BMSF can be broadly categorised into several approaches: functionalisation with bioactive molecules and optimising the mechanical cues of BMSF products to cells.

#### 3.6.1. Biochemical Functionalisation

The cornea’s transparency is essential for its role in focusing light onto the retina, and biomaterials intended for corneal repair must replicate its optical and mechanical properties. Ghosh et al. intended to improve tissue adhesion by incorporating dopamine into BMSF hydrogels, functionalised with limbus MSCs and a decellularised corneal matrix to improve corneal inductivity. The resulting construct showed high transparency and maintained desirable stiffness without inducing β-sheet formation, indicating that dopamine did not compromise the BMSF’s optical features. Interestingly, the key proteoglycans Lumican and keratocan, which are associated with corneal transparency, were expressed by limbus MSCs irrespective of the presence or absence of dopamine, suggesting dopamine contributes primarily to adhesive performance rather than providing additional biological cues relevant to corneal regeneration [267]. This multifunctional design leverages BMSF’s structural stability while utilising dopamine’s catechol groups for covalent bonding, representing a significant advance in corneal regeneration strategies. In contrast, BMSF and taurine composite scaffolds showed an average transmittance of 80% and above at 400 nm–650 nm compared to 88–90% of the human cornea, further advancing the transparency of BMSF [268]. Additionally, BMSF composited with polyacrylamide for mechanical property improvement and free radical crosslinking demonstrated good optical properties, great cell viability, and controllable degradation. Higher concentration BMSF–Polyacrylamide hydrogel showed a greater number of and more organised human corneal stromal cells with increased expression of extracellular matrix markers, indicating the inherent biocompatibility of BMSF for stromal repair [269]. This provides a directly scalable technical route for clinical corneal stromal repair. However, again, the lack of in vivo analysis compromises its potential clinical translatability, leaving its long-term features in biocompatibility unanswered. In addition, the cornea can also be anti-apoptotic with glial-cell-derived neurotrophic factor (GDNF) to be made into BMSF films. In vitro, the keratocyte cell viability assay showed that cell proliferation was benefited by GDNF at 250–500 ng/mL, with less cell death and apoptosis compared to the BMSF-only group [270]. In vivo assays showed that at the epithelial stromal damage site, smaller defect areas were observed in all silk groups regardless of GDNF concentration, with an increased level of proliferation factor B cell lymphoma-2 (Bcl-2) and a decreased level of pro-apoptotic factor [270]. Since Bcl-2 is a proliferation factor that has been proven to be associated with angiogenesis, the effect of GDNF on upregulating Bcl-2 has to be further studied, as angiogenesis compromises corneal transparency. The other factor contributing to the transparency of the cornea is the lack of blood vessels. Electrospun BMSF scaffolds were loaded with epigallocatechin gallate. In vitro results showed great cell compatibility of the functionalised scaffolds while inhibiting angiogenesis, as shown by significant inhibition of endothelial cell proliferation at days 4 and 6 [271]. Similarly, BMSF nanoparticles loaded with metformin have shown promising results in minimising the amount of neovasculature in vivo, highlighting that the controlled and localised release of metformin is a therapeutic approach to assist corneal regeneration [272]. Together, these studies suggest that successful BMSF-based artificial corneal scaffolds possess inherent transparency and should promote organised keratocyte proliferation while inhibiting angiogenesis to meet the fundamental requirements of corneal implants. None of the above studies have addressed the role of inflammation in altering keratocyte phenotypes, a key barrier to effective corneal repair. Addressing this gap, Zhang et al. investigated the efficacy of BMSF hydrogels loaded with an NF-κB inflammatory pathway inhibitor in preserving the phenotype of keratocytes. Results found that loss of phenotype-specific expression markers of keratocytes caused by IL-1β can be prevented by its inhibition in vitro, leading to an improved healing response of the cornea in a corneal defect model in vivo [273]. This highlights the critical role of inflammation control in maintaining keratocyte function and improving regenerative outcomes.

Despite advances in functionalisation and anti-angiogenic strategies, most studies lack in vivo validation and demonstrate insufficient control over inflammation-driven keratocyte phenotype loss. Manipulating scaffold architecture and mechanical properties offers a promising pathway to bridge these gaps by simultaneously supporting cell integration, regulating immune responses, and preserving corneal transparency.

#### 3.6.2. Topographical and Mechanical Cues in Corneal Regeneration

**Figure 11:**
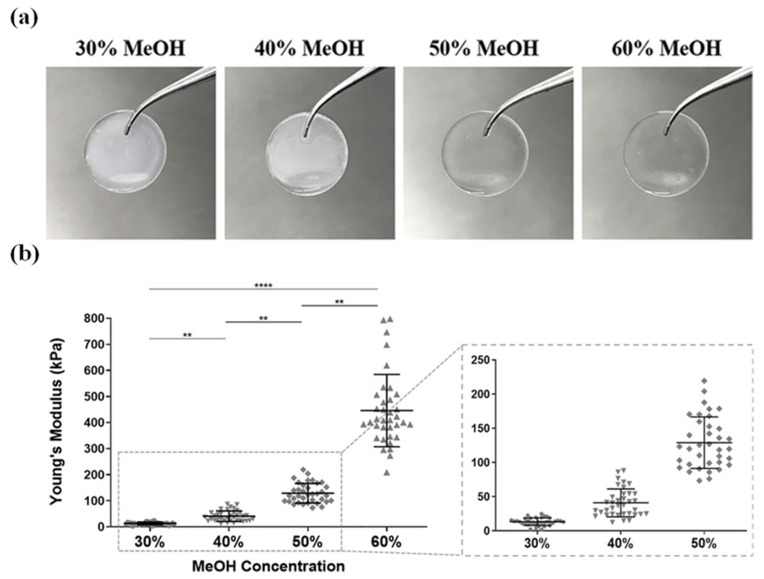
(**a**) Representative images of BMSF films crosslinked with different concentrations of annealing methanol. (**b**) Quantification of BMSF films’ stiffness in Young’s modulus shows that the stiffness of BMSF films was proportional to the concentration of ethanol. (mean ± SD, ** *p* < 0.01, **** *p* < 0.0001). Reprinted with permission from Ref. [275]. Copyright 2021, John Wiley and Sons.

**Figure 12:**
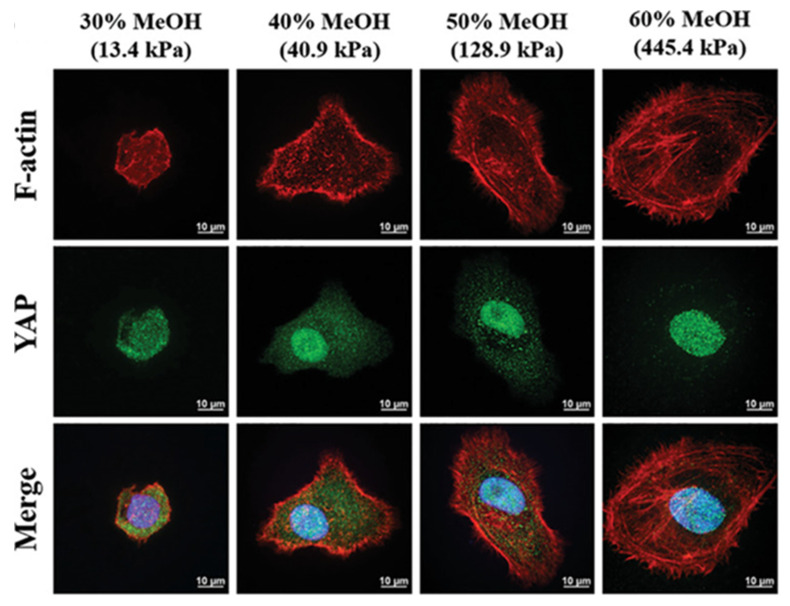
Immunofluorescence staining of YAP (green) in corneal epithelial cells cultured on BMSF scaffolds with various stiffnesses. YAP translocated to the nucleus as the stiffness of the scaffolds increased. Reprinted with permission from Ref. [275]. Copyright 2021, John Wiley and Sons.

Electrospinning techniques have also been employed to optimise both mechanical alignment and cellular compatibility. Studies investigating varying ratios of PCL and BMSF in electrospun composites found that higher BMSF content, particularly at 60:40 and 50:50 ratios, improved fibre alignment, optical transmittance, tensile strain, and cell viability. These formulations matched the mechanical and optical window of native cornea, indicating that mid-range BMSF proportions provide an optimal balance of transparency, biomechanical strength, and cellular compatibility for stromal substitution [274]. In addition to fibre alignment, BMSF blended with polyacrylamide (PA) exhibited a range of porosities depending on the ratio composition of each biomaterial. Scaffolds with a higher porosity (86%) demonstrated a higher level of keratocyte migration, proliferation, and related genes, as the scaffold architecture supported cell adhesion [269]. Additionally, Luo et al. designed collagen-coated BMSF scaffolds with nano grooves in different dimensions. Results demonstrated that corneal epithelial cell cultures on scaffolds with smaller grooves exhibited increased cell spreading, migration, and viability in vitro owing to the surface topography-induced focal adhesion and filopodia increase [100]. Beyond topography, matrix stiffness has emerged as a key regulator of corneal epithelial cell behaviour. BMSF scaffolds crosslinked in different concentrations of ethanol resulted in different stiffnesses, with higher methanol concentrations resulting in a higher Young’s modulus (Figure 11b). Results showed that the stiffness was proportional to methanol concentration and demonstrated stiffness-dependent cell spreading, with stiffer matrices promoting larger cell areas (Figure 12) [275]. YAP, a transcription factor that regulates cell survival as part of the HIPPO pathway, can translocate to the nucleus upon activation. Immunostaining showed a significant increase in the proportion of YAP in the nucleus compared to the cytoplasm on stiffer BMSF scaffolds [275]. Thus, regulating the stiffness of BMSF scaffolds is an essential factor in corneal cell regeneration, as cells are mechanosensitive. Together, these findings underscore the importance of tuning both the biochemical composition and mechanical properties of BMSF scaffolds to direct cell behaviour and enhance corneal regeneration.

#### 3.6.3. Clinical Translation Considerations and Future Directions

Taken together, existing studies demonstrate that BMSF offers a promising foundation for artificial corneal substitutes owing to its intrinsic transparency, tuneable mechanics, and favourable cytocompatibility. There are several challenges remaining for clinical translation. The optical clarity of BMSF is highly sensitive to its β-sheet content, which can increase during sterilisation, drying, or crosslinking, potentially causing long-term refractive inconsistency [276]. Moreover, the native cornea comprises three distinct cell layers, epithelial, stromal, and endothelial layers, each with unique biological requirements [277]. An effective BMSF corneal substitute must therefore not only support the proliferation of these cell types but also guide their spatial organisation to prevent functional loss. Therefore, future work should prioritise the development of composite, multilayer BMSF architectures that more closely replicate the hierarchical structure of the native cornea and maintain stable optical and biological performance over time.

## 4. Current Clinical Research Challenges and Perspectives on Silk Fibroin

BMSF has been used clinically as a surgical suture material for decades. Early silk sutures, which retained sericin, were suboptimal because sericin can elicit significant inflammatory responses [278]. Modern silk suture products, such as PERMA-HAND™ (Ethicon), are based on highly purified fibroin and therefore exhibit improved biocompatibility. However, these sutures are commonly coated with wax to reduce fraying, which has been associated with local inflammatory reactions [279]. More recently, commercial BMSF-based products with even higher purity and improved processing have been developed. SERI^®^ Surgical Scaffold, used primarily as a soft tissue support material, has demonstrated favourable immunogenicity, mechanical strength, and biodegradability. Its performance suggests potential applications in procedures such as hernia repair and reinforcement in breast reconstruction [280]. Similarly, Silk Voice^®^ is a BMSF augmentation material used for vocal-fold insufficiency. It has shown excellent longevity and low immunogenicity, making it a viable commercial option [281]. In dentistry, SimplySilk^®^, a multilayer BMSF membrane, is used to support guided bone regeneration in dental implant procedures. Its animal-free origin reduces the risk of pathogen transmission while providing an extended and predictable degradation profile. BMSF has proven to be one of the most versatile natural biomaterials in regenerative medicine owing to its unique balance of mechanical strength, biocompatibility, biodegradability, and processability. Yet, despite decades of progress from molecular characterisation to preclinical testing, the clinical translation of BMSF-based constructs remains limited. The current challenges lie not in demonstrating promise but in transforming that promise into predictable, scalable, and regulatory-ready technologies.

A major bottleneck originates at the earliest stage of production. The mechanical and biochemical quality of BMSF is influenced by silkworm diet, environmental conditions, and sericulture practices [282]. Batch-to-batch variability in cocoon quality translates into inconsistencies in molecular weight distribution and crystallinity, ultimately affecting scaffold reproducibility. Establishing standardised sericulture and cocoon-processing protocols, alongside molecular fingerprinting for quality assurance, is therefore fundamental for reliable clinical-grade silk production [283]. From the sericulture perspective, meaningful standardisation will require a transition from traditional silkworm farming to scientifically controlled sericulture systems, in which temperature, humidity, diet composition, and pathogen exposure are precisely regulated [284,285]. Selective breeding aimed at reducing genetic variability could further stabilise silk quality [284]. Additionally, modern machine-learning quality-control pipelines hold strong potential for reducing batch-to-batch variability in silk fibroin by enabling high-throughput quantitative assessment of key parameters such as molecular weight distribution, β-sheet crystallinity, and purity [286]. Machine learning has already demonstrated substantial value with potential direct adaptations to BMSF.

Processing and fabrication also present critical hurdles. Conventional degumming methods using alkaline or enzymatic solutions often degrade the fibroin’s peptide chains and reduce molecular weight, compromising mechanical performance and cell–material interactions [287]. Development of mild, non-denaturing extraction techniques, potentially integrating enzymatic selectivity, ionic liquids, or green solvent systems, would help preserve molecular integrity while eliminating sericin contaminants. For example, lactic acid/choline chloride deep eutectic solvents, derived from biomass, provide a far more biologically compatible option for silk dissolution and processing compared with lithium bromide [288]. In addition, solvents such as calcium chloride–ethanol–water can maintain silk fibroin in an extended random-coil conformation while rapidly dissolving high-molecular-weight BMSF under relatively mild conditions, enabling more efficient downstream processing and reducing protein damage [289]. Likewise, scaling fabrication techniques from laboratory to industrial levels remains problematic. Electrospinning, though widely used, offers limited throughput and architectural precision [252]. Emerging solvent-free fabrication methods, such as melt electrowriting (MEW), enable exceptional structural precision and design flexibility. However, BMSF itself lacks the thermoplastic behaviour required for MEW and therefore cannot be directly processed using this technique. To overcome this limitation, recent strategies combine BMSF with a thermoplastic MEW lattice, such as PCL, to integrate the geometric precision of MEW and the biocompatibility of BMSF [290]. In contrast, solution electrowriting only processes the BMSF solution at room temperature, avoiding thermal degradation while preserving native protein structure. However, solution electrowriting generally provides lower stacking accuracy because fibre deposition depends on water evaporation, which is difficult to control with high spatial precision [291]. Micro stereolithography can also achieve high spatial control but still requires optimisation for throughput and regulatory approval [292]. Achieving manufacturing consistency while maintaining fine structural control will be decisive for clinical translation.

Another challenge is the incomplete understanding of long-term in vivo behaviour. Most studies focus on short-term tissue responses in small animals, leaving degradation kinetics, metabolic by-products, and systemic immune effects poorly characterised [293]. Although BMSF is generally regarded as biocompatible, its degradation products, primarily peptides and amino acids, may interact differently across organs or in disease states [294]. Large-animal and chronic studies, accompanied by advanced imaging and metabolomic tracking, are required to define safe degradation windows and inform scaffold design. Moreover, the immune response to BMSF is highly context-dependent [83]. Architectural cues such as stiffness, porosity, and surface chemistry influence macrophage polarisation and foreign body reaction. Overcoming the limitations of in vivo models in long-term investigations of BMSF organoid systems has gained significant attention. These platforms recapitulate the physiological, structural, and functional features of native tissues. Recent work has shown that BMSF is highly compatible with organoid engineering, particularly through its use in fabricating adaptive, stimuli-responsive, and long-term stable scaffolds [21]. BMSF organoid platforms allow for controlled, extended investigations of BMSF biodegradation, immune interactions, and cellular responses that are difficult to capture in conventional animal models. Therefore, systematic immune-profiling frameworks that integrate both local and systemic responses should become part of preclinical testing.

Finally, translational research must bridge materials science with regulatory and clinical realities. Quality control standards for BMSF are not yet harmonised across countries, and scalable sterilisation, storage, and sterilant residue validation remain unresolved. Moreover, sterilisation can significantly alter the molecular structure and functional performance of BMSF biomaterials. Physical methods such as autoclaving with high-temperature steam have been shown to increase β-sheet content while narrowing molecular weight distribution, resulting in stiffer scaffolds [295,296]. In contrast, gamma radiation disrupts the BMSF molecular structure by diminishing the molecular weight and chain scission, thereby promoting degradation in vivo [297,298,299]. Chemical sterilisation approaches also affect BMSF differently: ethanol can increase β-sheet content while largely preserving scaffold integrity, whereas ethylene oxide maintains both β-sheet content and mechanical properties but exhibits high cytotoxicity, significantly reducing cell proliferation [296,300,301]. Overall, the choice of method must balance molecular stability with biocompatibility.

Collectively, collaboration between materials scientists, clinicians, and industry partners is essential to establish Good Manufacturing Practice pipelines, define biocompatibility standards, and accelerate first-in-human trials. The next phase of silk-based innovation will depend less on inventing new formulations and more on standardising, validating, and integrating existing advances into reliable clinical workflows.

## 5. Conclusions

BMSF has evolved from an ancient textile fibre into a modern platform for regenerative medicine. Its combination of structural versatility, biocompatibility, and molecular tunability has enabled remarkable progress across tissue types from bone, cartilage, and skin to neural, vascular, and corneal regeneration. Yet the field now stands at a turning point: success will hinge on how effectively we translate molecular precision and laboratory creativity into reproducible, safe, and clinically effective therapies.

The next frontier lies in integration—integrating disciplines, length scales, and biological insights. Advances in nanoscale fabrication, electrowriting, and bio-printing have enabled the coupling of BMSF’s hierarchical structure with instructive topographies and biochemical patterning that directly communicate with cells. Parallel developments in bioinformatics, machine learning, and organ-on-chip systems promise predictive control over degradation, immune modulation, and cell behaviour. By combining these technologies, BMSF can transition from a passive scaffold to an active instructive matrix that guides tissue repair through dynamic feedback between material and biology.

Clinically, the near future will likely see BMSF’s most immediate impact in applications that demand both strength and biocompatibility, such as corneal substitutes, vascular grafts, and bone fillers, where its mechanical reliability and tuneable degradation properties outperform many synthetic polymers. Long term, silk’s potential extends further: as a platform for smart implants capable of controlled drug delivery, biosensing, or transient electronic interfaces that safely dissolve after use. These prospects exemplify silk’s capacity not merely to replace tissue but to redefine how biomaterials interact with living systems.

In summary, BMSF represents more than a biomaterial. It is an adaptable, intelligent system whose future lies in convergence. The continued evolution of processing precision, immune-responsive design, and interdisciplinary collaboration will determine whether the promise of silk becomes a clinical reality. With strategic investment in translational science and standardisation, BMSF could move from the bench to the bedside as a cornerstone material for the next generation of regenerative and bio-integrated medical technologies.

## Figures and Tables

**Table 1:** Comparison between mulberry silk fibroin and non-mulberry silk fibroin.

Feature	Mulberry Silk Fibroin (BMSF)	Non-Mulberry Silk Fibroin
Natural cell adhesion motif (RGD)	Absent [26].	Present. Enhancing integrin-mediated cell adhesion and proliferation [22,23,24].
Processing reproducibility and standardisation	Established and mature preparation procedure. Reproducible batch to batch properties [27].	Varies across species. Require species-specific optimisation [17,25,27].
Mechanical properties and structural stability	Beta sheet content can be tuned to meet the mechanical needs of specific tissues. Higher stiffness [7,8].	Some non-mulberry silk fibroin offers higher extensibility and lower stiffness [18].
Immunogenicity	Low immunogenicity with mild inflammatory response and few foreign body cells [28,29].	Low immunogenicity with mild inflammatory response [29].
Availability and scalability	Mulberry silk is mass-produced with easy access [30].	Less produced industrially, difficulty in minimising variation between batches and quality standardisation [30].

**Table 4:** Examples of BMSF-based biomaterials for cartilage regeneration.

BMSF-Based Biomaterial Form	Biomaterial Composition/Modification	Key Findings
Scaffold (Sponge)	Porous scaffold	The scaffold promoted adhesion, proliferation, and glycosaminoglycan deposition, with increased expression of chondrogenic differentiation markers in adipose stem cells (ASCs) in vitro and enhanced tissue ingrowth in a subcutaneous model by day 28 [118].
Hydrogel	Montmorillonite	The hydrogel improved chondrogenic differentiation of BMSCs and promoted osteochondral regeneration in a rabbit femoral defect model at 12 weeks [119].
Scaffold (3D printed)	Hyaluronic acid and BMSC affinity peptide	BMSC recruitment with BMSF scaffolds improved the outcome of cartilage healing in rabbit cartilage defects at 12 weeks, with high biocompatibility compared to scaffolds without BMSC affinity peptides [120].
Hydrogel	BMSF was modified with methacrylate and then loaded with kartogenin	Modified BMSF scaffolds, both with and without kartogenin, increased ICRS scores compared to the negative control; the presence of kartogenin further enhanced chondrogenic differentiation without affecting cell viability [121].
Scaffold (3D printed)	BMSF was modified with methacrylate and then loaded with an antioxidant (methacrylate-modified rutin)	The reduced oxidative stress was associated with the upregulation of collagen-related factors and downregulation of matrix degradation factors, resulting in the enhancement of cartilage tissue regeneration [122].
Scaffold (Lyophilised)	Decellularised cartilage extracellular matrix	The addition of the decellularised cartilage matrix and the porous structure produced from lyophilisation synergistically promoted chondrogenic differentiation, as indicated by upregulated cartilage-related markers [123].
Hydrogel	Chitosan, TGF-β1, BMSCs	The hydrogel sustainably released TGF-β1, which led to increased expression of chondrogenic markers of BMSCs and improved cartilage regeneration in a rat cartilage defect model [124].

**Table 5:** Examples of BMSF-based biomaterials for bone regeneration.

BMSF-Based Biomaterial Form	Biomaterial Composition/Modification	Key Findings
Hydrogel	Piezoresistive mxene	The hydrogel induced Ca^2+^/CALM signalling and M2 macrophage polarisation, resulting in significant bone regeneration at 12 weeks in rat cranial defects [151].
Harversian canal mimicking hydrogel	Increased β-sheet crystallite and hydroxyapatite	The hydrogel increased mineralisation efficiency by 3.3 fold, enabling successful repair of load-bearing femoral condyle defects within 1 month in rabbits [152].
Scaffold (sponge)	Strontium hydrogen phosphate, ginsenoside Rg1, gelatine	The scaffold promoted osteogenic differentiation of BMSCs, inhibited osteoclastogenesis, stimulated angiogenesis, and improved bone repair in critical sized cranial defects at 12 weeks through the TLR-4/P13K/Akt signalling pathway [153].
Scaffold (electrospun)	Polycaprolactone-BMSF composite scaffold with strontium carbonate	The scaffold improved cell adhesion and increased ALP activity and mineralisation in vitro [154].
Scaffold (sponge)	Graphene oxide	The scaffold enhanced osteogenic activity of BMSCs, showing higher ALP activity and osteogenic protein expression within 21 days [155].
Scaffold (freeze-dried)	Enzyme crosslinked, then loaded with hydroxyapatite	The scaffold demonstrated increased mechanical strength and promoted osteoblastic differentiation and growth [156].
Hydrogel	Graphene oxide and alginate	The hydrogel induced M2 macrophage polarisation via NF-κB and MAPK pathways, enhancing osteogenic and mineralisation in distal femoral defects in rats [157].
Scaffold (sponge)	Hydroxyapatite, polylactic-co-glycolic acid microspheres loaded with naringin	The scaffold promoted osteogenic differentiation of BMSCs and enhanced bone regeneration in a rabbit femoral distal bone defect model at 6 weeks via the Notch signalling pathway [158].

**Table 6:** Examples of BMSF-based biomaterials for skin healing.

BMSF-Based Biomaterial Form	Biomaterial Composition/Modification	Key Findings
Scaffold (electropsun)	Soy protein isolate	Fibroblast proliferation (L929-RFP) was enhanced on scaffolds with higher soy protein isolate content. The scaffold achieved complete (≈100%) wound healing in a rat wound model within 14 days [179].
Hydrogel	-	The BMSF-only hydrogel recruited a higher proportion of regenerative macrophages, which correlated with reduced scar length [180].
Dressing (electrospun)	Quaternised chitin	The dressing promoted cell proliferation in vitro and accelerated wound healing, increasing number of hair follicles and blood vessels by day 15 [181].
Scaffold (sponge)	Anti-inflammatory peptide-1, vertically aligned cryogel fibres	The scaffold reduced inflammation as indicated by increased CD-206 expression, enhanced angiogenesis, restored epithelial thickness, and promoted collagen. deposition at day 21 in diabetic rats [182]
Scaffold (electrospun)	Titanium dioxide, gelatine, polycaprolactone	High cell viability and antibacterial properties were observed; in vivo, the scaffold achieved nearly complete wound closure within 28 days [183].
Scaffold (sponge)	Fibrinogen, hyaluronic acid	The scaffold enhanced epithelial regeneration, dermal remodelling, and angiogenesis, resulting in accelerated wound healing [184].
Hydrogel	Polyvinylpyrrolidone, silicotungstic acid	The hydrogel exhibited strong adhesiveness, rapid self-healing, and antibacterial activity, leading to faster wound area reduction [185].
Hydrogel	Neurotrophin-3	The hydrogel accelerated wound healing by increasing collagen III deposition via the tropomyosin receptor kinase C downstream pathway [186].

**Table 7:** Examples of BMSF-based biomaterials for nerve regeneration.

BMSF-Based Biomaterial Form	Biomaterial Composition/Modification	Key Findings
Hydrogel	Magnesium ions, bisphosphonate-modified alginate	The hydrogel regulated macrophage polarisation and promoted neurite growth and myelin sheath regeneration, resulting in functional motor recovery in rat sciatic defects at week 8 [207].
Scaffold (electrospun)	Methanol treatment post-electrospinning	The scaffold facilitated neurite growth, increased sciatic functional index, cross-sectional area, and myelin thickness in rat sciatic nerve defects at week 12 [208].
Scaffold (sponge)	Calcium titanate nanoparticles	Optimal calcium titanate concentration stimulated Schwann cell proliferation and attachment while preserving cell function in vitro [209].
Hydrogel	Black phosphorus, glycyrrhizic acid	The hydrogel reduced oxidative damage, promoted M2 macrophage polarisation, induced neural stem cell differentiation, restored signal conduction at the spinal cord injury site, and improved motor function in mice with spinal cord hemisection at week 6 [210].
Scaffold (lyophilised)	Nerve growth factor and glial-cell-line-derived neurotrophic factor	The scaffold promoted motor innervation and increased the number of myelinated neurons at the defect site, achieving recovery comparable to autologous grafts [211].
Scaffold (electrospun)	Polylactic acid and exosomes derived from human endometrial stem cells	The scaffold enhanced axonal regeneration, neovascularisation, and functional recovery in rat sciatic nerve defects, with outcomes comparable to autografts at week 12 [212].
Scaffold (electrospun)	Polylysine	Aligned scaffolds achieved complete biodegradation in 4 weeks and promoted sciatic nerve regeneration, showing functional and electrophysiological recovery comparable to autografts without scarring at week 12 [213].

**Table 8:** Examples of BMSF-based biomaterials for endothelisation and vascularisation.

BMSF-Based Biomaterial Form	Biomaterial Composition/Modification	Key Findings
Vascular graft (knitted)	-	Five out of six silk vascular grafts maintained high patency with complete endothelial coverage at 3 months post implantation [238].
Vascular graft (knitted)	Elastin	All vascular grafts remained patent at 2 weeks with endothelial cell coverage [239].
Vascular graft (electrospun)	Glycerol	Grafts matched native vessel compliance and achieved full endothelial coverage after 5 days in vitro [240].
Vascular graft (casting)	Elastin-like recombinamers	Achieved compliance matching native vessels with low thrombogenicity [241].
Vascular graft (knitted)	IKVAV and REDV peptides via transgenic silkworms	The vascular graft enhanced endothelialisation and selective cell adhesion without increased platelet adhesion [242].
Vascular graft (woven)	Inner electrospun polycaprolactone-collagen layer	Improved mechanical robustness in burst pressure, suture retention, and compliance with enhanced endothelial cell adhesion [243].

**Table 9:** Examples of BMSF-based biomaterials for corneal regeneration.

BMSF-Based Biomaterial Form	Biomaterial Composition/Modification	Key Findings
Hydrogel (film)	Gelatine methacrylic	Supported the attachment, proliferation, and upregulated metabolism of stromal cells, achieving 97% coverage of stromal cell within 5 days [262].
Hydrogel	Hyaluronic acid	Medium- and high-molecular-weight BMSF hydrogels upregulated key corneal repair genes in vitro and accelerated wound closure in mice [263].
Scaffold (electrospun)	Gelatine	Epithelial regeneration was associated with the pore size of the scaffold [264].
Scaffold (film)	Curcumin	Enhanced roughness, transparency, and hydrophilicity; increased corneal endothelial cell-specific mRNA at lower curcumin concentrations [265].
Scaffold (film)	Gelatine	Supported cell adhesion, viability, proliferation, and differentiation; upregulated keratocyte markers and promoted collagen I deposition and ECM formation, mimicking native corneal structure [266].

## Data Availability

This review does not include any original primary research data, software, or code, and no new data were generated or analysed in its preparation.

## Abbreviations

The following abbreviations are used in this manuscript:

BMSF	*Bombyx mori* silk fibroin
RGD	Arg-Gly-Asp
FBR	Foreign body response
ePTFE	Expanded polytetrafluoroethylene
TGF-β	Transforming growth factor-β
IL-1β	Interleukine-1β
TNF-α	Tumour necrosis factor-α
NF-κB	Nuclear factor-κB
YAP	Yes-associated protein
PCL	Polycarpolactone
ICRS	Cartilage Repair Society scoring system
BMSC	Bone mesenchymal stem cells
DCM	Decellularised cartilage matrix
ROS	Reactive oxygen species
IL-4	Interleukine-4
CNS	Central nervous system
PNS	Peripheral nervous system
NSC	Neural stem cell
NIH	Neointimal hyperplasia
VSMC	Vascular smooth muscle cells
HFIP	Hexafluoroisopropanol
MSC	Mesenchymal stem cell
GDNF	Glial-cell-derived neurotrophic factor
MEW	Melt electrowriting
GelMA	Gelatine methacrylic
MPA	Methylprednisolone acetate
Bcl-2	B-cell lymphoma-2
